# Large Yellow Croaker (*Pseudosciaena crocea,* Richardson) E2F4, a Cyclin-Dependent Transcription Factor, Forms a Heterodimer with DP1

**DOI:** 10.3390/ijms26115343

**Published:** 2025-06-02

**Authors:** Xiaohui Cai, Honglin Chen, Jing Fang, Meijuan Xu, Meijuan Chen, Qiancheng Qi, Peng Xu, Patrick C. Hanington, Xinzhong Wu

**Affiliations:** 1 Guangxi Key Laboratory of Beibu Gulf Marine Biodiversity Conservation, College of Marine Sciences, Beibu Gulf University, Qinzhou 535011, China; 2 Key Laboratory of Environment Change and Resources Use in Beibu Gulf, Ministry of Education, Nanning Normal University, Nanning 530001, China; 3 School of Public Health, University of Alberta, Edmonton, AB T6G 2R3, Canada

**Keywords:** large yellow croaker *Pseudosciaena crocea*, E2F transcription factors, subcellular localization, expression pattern, protein interaction

## Abstract

E2F transcription factors regulate cell cycle progression by influencing the expression of proteins required for the G_1_-S phase transition and DNA synthesis with its heterodimeric partners (DP1 or DP2). The dimerization domain is the E2Fs and DP1 protein interaction interface and is believed to function in protein dimerization. In this study, eight E2F transcription factors (PcE2F1–8) of large yellow croaker *Pseudosciaena crocea* and one dimerization partner (PcDP1) are identified in the genome of large yellow croakers. The prediction of E2Fs conserved domains revealed that PcE2F1–6 has one DNA-binding domain (DBD) and one dimerization-binding domain (DD), while PcE2F7–8 only possess two duplicate DBDs but not DD, indicating that E2F7–8 cannot form the E2F/DP1 heterodimer. To explore whether PcDP1 is a partner of PcE2F1–6, the ORF of PcE2F1–6 was cloned. Subsequently, its sequence characteristics, the expression pattern in healthy fish, and subcellular co-localization were analyzed, and an interaction between PcDP1 and PcE2F1–6 were detected directly by yeast two-hybrid and BiFC. The *PcE2F1*, *PcE2F2*, *PcE2F3*, *PcE2F4*, *PcE2F5*, and *PcE2F6* genes encode a protein of 454, 448, 444, 392, 362, and 396 amino acids, respectively, with accession numbers QFZ93593.1, QFZ93594.1, QFZ93595.1, QFZ93596.1, QFZ93597.1, and QFZ93598.1, respectively. Sequence characteristics analysis found that PcE2F1–5 but not PcE2F6 proteins share the pocket protein-binding domain sequestering in dimerization domains and transactivation domains. The PcE2F1,2,4 proteins possess one nuclear localization signal (NLS), and PcE2F3 protein possess two NLSs, but there is no NLS in PcE2F5 and 6 protein. Moreover, PcE2F4 also contains one NES. However, PcE2F1–6 proteins were all located in nucleus by using Euk-mPloc 2.0 programs and were confirmed by performing the Cherry and EGFP reporter assay. Regarding co-expression of DP1, only E2F4 can transfer DP1’s subcellular location from cytoplasm to the nucleus. RT-qPCR analysis indicated that PcE2F1–6 are constitutively and tissue specifically expressed in all of the tissues tested of a healthy large yellow croaker. The *PcE2F1*–*6*, except for *PcE2F3*, mRNA levels were all detected higher in the liver. *PcE2F1*–*4* were also highly specifically expressed in the kidney, *PcE2F4*,*6* in the brain, and *PcE2F5* in the spleen of a healthy large yellow croaker, respectively. Using a yeast two-hybrid system, PcE2F4 interacting with PcDP1 was identified. The interaction between PcE2F4 and PcDP1 was further confirmed by a bimolecular fluorescence complementation (BiFC) assay. Collectively, these results indicate that an interaction between PcE2F4 and PcDP1 was detected, which may form heterodimer E2F4/DP1 to regulate cell cycles and immune-related pathways in large yellow croakers.

## 1. Introduction

Cell cycle regulation is critical to normal growth and division. The cell cycle checkpoints, particularly the G_1_toS checkpoint, serve crucial roles in the transmission of genetic information and cell replication. Central to the temporal control of the G1toS phase transition is the regulation of the activity of the E2F transcription factor (TF) family [1,2]. In mammalian cells, the E2F transcription factors constitute a superfamily of basal elongation factors, which comprises eight different proteins (E2F1–8) and its dimerization partner (DP) proteins (DP1–4) [1,3]. Based on their function, they have been categorized as activators (E2F1–E2F3a) and repressors (E2F3b–8) [4]. At the early G1 phase of the cell cycle and in quiescent cells, the E2F4 and E2F5 transcriptional repressors interact with the retinoblastoma protein (RB) to inhibit target gene transcription [5], while transcriptional activators (E2F1–3) with free RB replace the repressors, leading to the increase in G1/S phase gene expression and promote DNA replication at the S phase [6]. During progression from quiescent to S phase, RB phosphorylation by cyclin-CDK complexes activates E2F activity [7]. E2F6–8 negatively regulates the transcription of E2F-responsive genes [8,9,10]. Besides their cell cycle regulation, E2Fs also regulate genes involved in oncogenesis, organ development, nerve regeneration, cell proliferation, and apoptosis. For example, E2F-1 regulates apoptosis as well as proliferation, in part by stabilizing the p53 tumor suppressor, which is an important mediator of apoptosis [11]. Elevated expression levels of E2F4 have been associated with gastric cancer [12], solid osteosarcoma [13], and breast tumor progression [14]. In addition, E2F4 can partially promote neuronal regeneration and functional recovery after spinal cord injury in zebrafish [15], and E2F5 is required for normal development of the brain, eye, and ovarian of zebrafish [16].

On the other hand, E2F proteins have also been categorized as classic E2Fs (E2F1–6) and atypical E2Fs (E2F7–8) according to their conserved domains [9]. Studies revealed that six classical E2F proteins all possess one N-terminally located DNA-binding domain (DBD) immediately followed by a dimerization domain, allowing interaction with a DP protein [2], while atypical E2Fs have only two DNA-binding domains (DBD1 and DBD2) but no dimerization domain [4]. The three DP family members, DP1, DP2/3, and DP4, encode proteins that are related to the E2F family with homology in the DNA-binding and dimerization domains of E2F [17]. Although recent research has verified that DP proteins can directly associates with other proteins (i.e., p53) [18], the DP proteins mainly play a role as the heterodimeric partners for the E2F family to increase the DNA-binding efficiency and transactivation potential of the E2F proteins. E Huber et al. (1993) found that a dramatic (100- to 1000-fold) increase in specific DNA-binding activity of E2Fs was observed on mixing the E2Fs and DP1/2 components together [19]. It is well established that the classical E2F transcriptional activity is mediated by heterodimers of E2F1–6 with DP1 or DP2 proteins through their dimerization domain [17,20]. The dimerization of E2F/DP is a prerequisite for high-affinity, sequence-specific binding of the E2F proteins to promoters of target genes [21]. Now, studies have showed that DP proteins not only affect the phosphorylation state of E2F/DP heterodimer but also affect the types of proteins binding, subcellular localizations, and expression level of E2Fs, which all influence the DNA-binding ability and transcription activity of the E2F/DP heterodimer [22]. In embryos, the expression patterns of E2F target genes *RNR2* and *PCNA* are lost in *dDP* mutants [23,24]. Co-expression of E2F4 with DP2 protein can result in them transferring from the cytoplasm to the nucleus [25]. Of note, DP1 protein can also block E2F1 competing with p50 for binding to p65, which impairs the NF*κ*B signal and sensitizes cells to apoptosis [26]. Therefore, DP protein plays an important role in the regulation of E2F-dependent genes, DNA synthesis, apoptosis, and even tumorigenesis.

The large yellow croaker (*Pseudosciaena crocea* Richardson, 1864) is one of the most economically important marine fish species cultured in the coastal temperate zone, especially in the Fujian and Zhejiang provinces of China. In order to obtain important genes involved in physiological and pathological processes, we originally constructed the cDNA library of the head and kidney of a large yellow croaker with PG as immunostimulant in 2008 [27] (Huang et al., 2008), and subsequently the *DP1* gene (named as *PcDP1* with an accession number of DQ821446) was cloned from SSH cDNA library [27,28]. A comparison of Pc-DP1with human DP1 and house mouse DP1 indicated that their DNA-binding and dimerization domains are strikingly conserved [28], implicating the importance of these proteins in cellular function. And, we have confirmed the DNA-binding activity of PcDP1 with a DIG-labeled probe containing a consensus DNA-binding site using EMSA [28] (Cai et al., 2021). However, whether PcDP1 can form heterodimers with E2Fs of a large yellow croaker and with which one of the E2Fs has not been reported.

Presently, the genome of large yellow croakers has been sequenced in 2014 and 2015, successively [29,30]. Through searching the whole genome, we found that eight E2F transcription factors are present in different chromosomes of large yellow croakers *Pseudosciaena croce*, while its dimerization partner DP1, not DP2 and DP3, was found in the genome of large yellow croakers. To further explore whether PcDP1 is a heterodimer partner of PcE2F1–6, the classic E2Fs of large yellow croakers were cloned. Subsequently, their sequence structural characteristics, expression patterns, and subcellular localizations were analyzed. The interaction between PcDP1 and PcE2F1–6 was detected directly by yeast two-hybrid and BiFC. We show that *PcE2F1–6* were specifically expressed in the liver and kidney, and that the PcE2F4 protein can interact with PcDP1 and co-localize with it from the cytoplasm to nucleus, which lays the foundation for further study on the biological function in the cell cycle- and immune-related pathways of heterodimer E2F4/DP1.

## 2. Results

### 2.1. Identification and Cloning of Large Yellow Croaker E2Fs Family Genes

**Figure 1:**
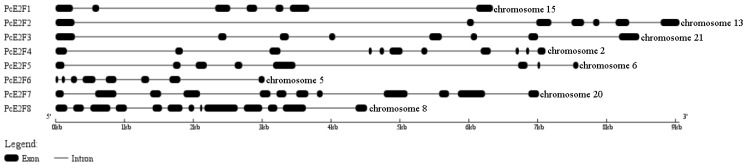
Structure of large yellow croaker E2F genes and its transcripts E2F1, E2F2, E2F3, E2F4, E2F5, E2F6, E2F7, and E2F8. The large yellow croaker E2F gene contains 7, 7, 8, 11, 8, 8, 12, and 13 exons and introns about 3~9 kb.

**Figure 2:**
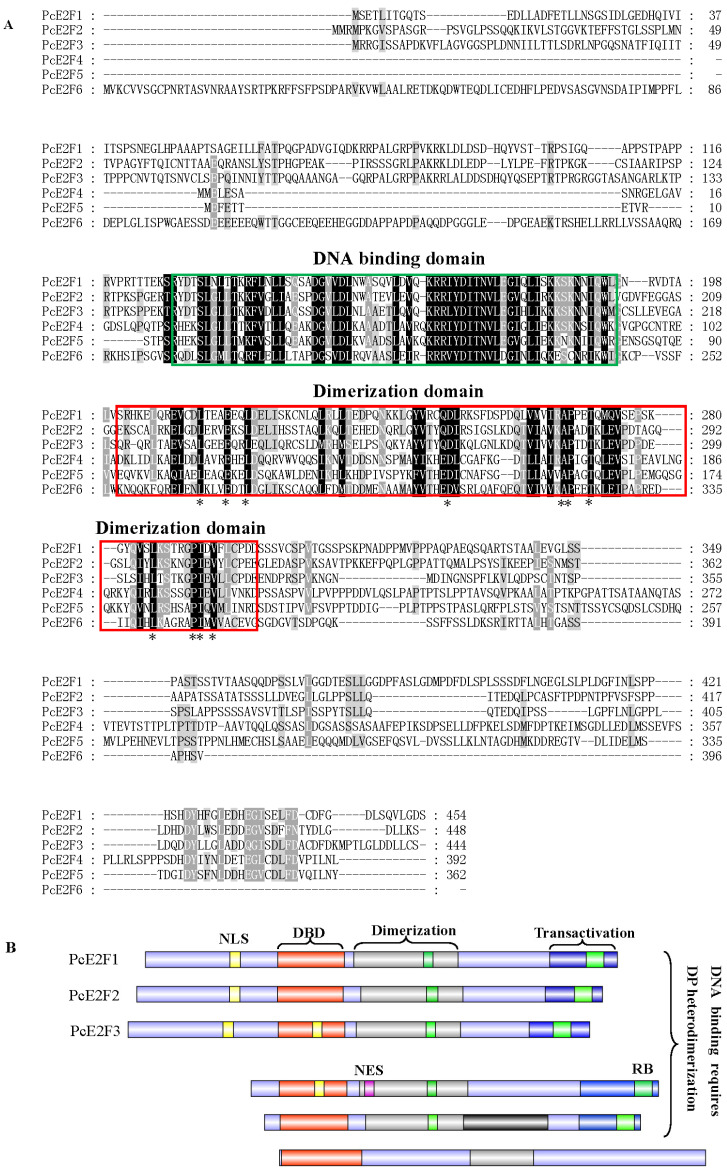
Sequence alignment of E2F family proteins in the large yellow croaker. (**A**) Multiple sequence alignment of E2F1–6 proteins. Sequence alignment was performed using Clustal X 2.0 and GeneDoc software 2.7. The functional domains were determined by searching the CDD database (https://www.ncbi.nlm.nih.gov/Structure/cdd/wrpsb.cgi, accessed on 11 July 2019). The asterisk indicates identical residues. (**B**) Schematic representation of the domain structure of full-length E2F1–8 proteins. Nuclear localization signals and leucine-rich nuclear export signals were predicted by cNLS Mapper (http://nls-mapper.iab.keio.ac.jp/cgi-bin/NLS_Mapper_form.cgi, accessed on 13 July 2019) and NetNES 1.1 Server (https://services.healthtech.dtu.dk/services/NetNES-1.1/, accessed on 13 July 2019). The DNA-binding, dimerization, transactivation domains, NLS, NES, and RB family protein binding domain (RB) are indicated by orange, gray, blue, yellow, pink, and green boxes, respectively.

Through searching the large yellow croaker genome database (https://www.ncbi.nlm.nih.gov/genome/?term=Larimichthys+crocea, accessed on 15 March 2019), we identified eight different E2F family genes, *E2F1* (XM_010743609.3), *E2F2* (XM_027275214.1), *E2F3* (XM_019256914.2), *E2F4* (XM_010734861.3), *E2F5* (XM_027288557.1), *E2F6* (XM_027279409.1), *E2F7* (XM_010750303.3), and *E2F8* (XM_027281565.1). Our results showed that they are located on different chromosomes, with *E2F1* being on chromosome 15, *E2F2* being on chromosome 13, *E2F3* being on chromosome 21, *E2F4* being on chromosome 2, *E2F5* being on chromosome 6, *E2F6* being on chromosome 5, *E2F7* being on chromosome 20, and *E2F8* being on chromosome 8. These contained 7~13 exons separated by 6~12 introns, respectively (Figure 1). The prediction of E2F family protein conserved domains revealed that E2F1–6 has one N-terminally located DNA-binding domain (DBD) and one dimerization-partner-binding domain (DD) following the DBD while E2F7–8 only possess two duplicate DBDs but no DD, which means that E2F7–8 cannot form the E2F/DP1 heterodimer (Figure S1). Therefore, the open reading frame (ORF) of six E2F genes (*E2F1–6*) were amplified from mixed cDNA of a large yellow croaker liver, spleen, intestines, kidney, head kidney, brain, gill, and heart tissues using specific primers designed based on the above predicted E2F genes sequences, and were named *PcE2F1*, *PcE2F2*, *PcE2F3*, *PcE2F4*, *PcE2F5*, and *PcE2F6,* respectively. The assembled *PcE2F2*, *PcE2F3*, *PcE2F4*, *PcE2F5*, and *PcE2F6* sequence was 1365 bp, 1247 bp, 1335 bp, 1179 bp, 1089 bp, and 1181bp in length, respectively, which has been deposited into the GenBank database with the accession numbers MK922550, MK922551, MK922552, MK922553, MK922554, and MK922555, respectively. The predicted ORF of *PcE2F2*, *PcE2F3*, *PcE2F4*, *PcE2F5*, and *PcE2F6* encodes, respectively, a protein of 454, 448, 444, 392, 362, and 396 amino acids with the accession numbers QFZ93593.1, QFZ93594.1, QFZ93595.1, QFZ93596.1, QFZ93597.1, and QFZ93598.1, respectively. The deduced E2F proteins of a large yellow croaker contains one DBD and one DD (Figure 2B), which are well conserved in large yellow croakers (Figure 2A) and among multiple species, including tilapia, zebrafish, human, mouse, and bovine (Figures S2–S7). In addition, PcE2F1–5 but not PcE2F6 proteins share the pocket protein-binding domain sequestering in the DD and transactivation domain, and it has been confirmed that it can specifically interact with distinct members of the pRB family proteins. Furthermore, PcE2F5 possesses a linker for the activation of T-cells (LAT) following the dimerization domain, indicating that it may be involved in the development of T-cells in the large yellow croaker.

**Figure 3:**
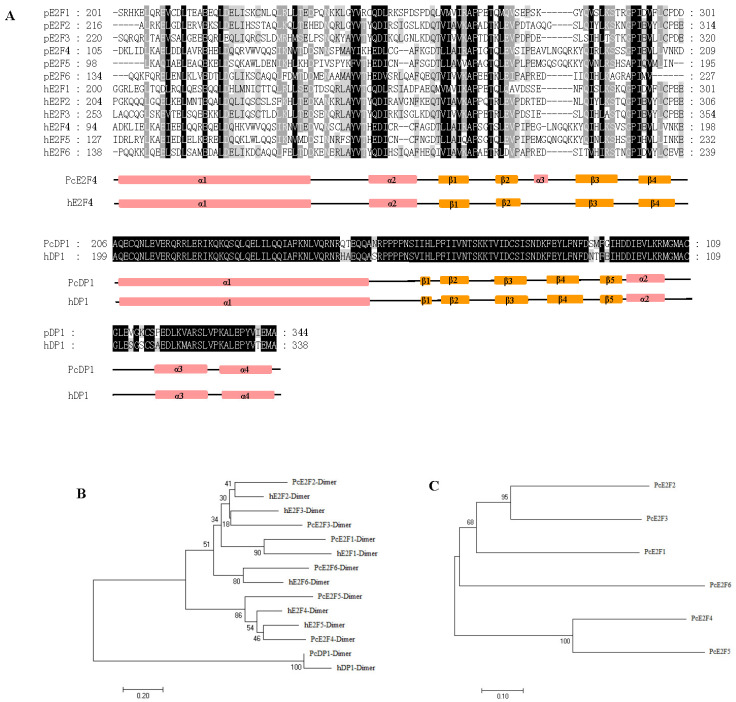
Sequence alignment and evolutionary relationship of dimerization domains between large yellow croakers and humans. (**A**) Sequence alignment of E2F and DP1 domains between large yellow croakers and humans. The secondary structure elements of PcE2F4 were predicted by the Psipred 4.0 software (http://bioinf.cs.ucl.ac.uk/psipred/, accessed on 20 July 2019). The secondary structure elements of hE2F4 were quoted from its crystal structure. Black indicates identical residues. Grey indicates highly conserved amino acids residues. Pink indicates a helix structure. Yellow indicates a strand structure. (**B**) The evolutionary relationship of E2F1–6 and DP1 dimerization domains between large yellow croakers and humans. hE2F1: human E2F1 (NP_005216.1), hE2F2: human E2F2 (NP_004082.1), hE2F3: human E2F3(NP_001940.1), hE2F4: human E2F4 (NP_001941.2), hE2F5: human E2F5 (NP_001942.2), hE2F6: human E2F6 (NP_937987.2), and hDP1: human DP1 (NP_009042.1). (**C**) The evolutionary relationship of E2F1–6 and DP1 of large yellow croakers. Phylogenetic analyses were performed using the neighbor-joining method in MEGA 7.0 with 1000 bootstrap replications.

To investigate the possibility of forming a heterodimer between PcE2F1–6 and PcDP1, the alignment of the dimerization domains of PcE2F1–6 and PcDP1 compared to human E2F1–6 and DP1 using the Clustal X program and GeneDoc software 2.7 shows high homology with DP and E2F intermolecular contacts motif (Figure 3A). Further analysis of the secondary structure elements of PcE2F4 and PcDP1 revealed that they both contain a varying number of helices and strands and are similar to human E2F4 and DP1 (Figure 3A). However, there is an extra helix (α3) in the secondary structure of PcE2F4. Phylogenetically, the dimerization domain of large yellow croaker PcE2F1–6 and human hE2F1–6, PcDP1, and hDP1 cluster together (Figure 3B). Significantly, when the full-length large yellow croaker E2F1–6 proteins were analyzed for evolutionary relationship on the basis of their primary structure, we found that PcE2F1, 2 and 3, and PcE2F4, 5, and 6 cluster together, which shows a segregation pattern that is reflective of their known functional characteristics (Figure 3C).

### 2.2. Subcellular Colocalization of Large Yellow Croaker E2F and DP1

**Figure 4:**
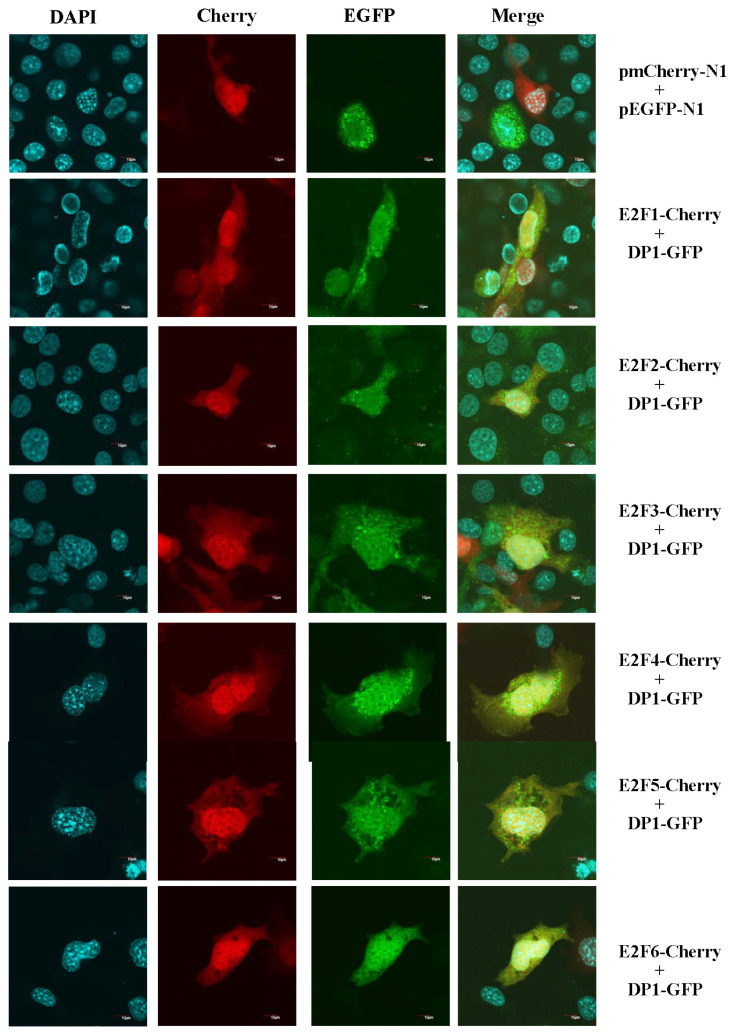
Subcellular colocalization of pE2Fs protein and pDP1 analyzed by a cherry and GFP reporter assay. COS-7 cells were co-transfected with cherry and GFP reporter constructs expressing a cherry-tagged E2Fs and EGFP-tagged DP1. Empty pmcherryN1 and pEGFPN1 vectors were used as a control. Nuclear DNA was stained with DAPI, and cells were analyzed with a fluorescence confocal microscope. Scale bars, 10 μm.

Using cNLS Mapper (http://nls-mapper.iab.keio.ac.jp/cgi-bin/NLS_Mapper_form.cgi, accessed on 13 July 2019) programs, the PcE2F1,2,4 proteins were predicted to possess one nuclear localization signal (NLS) and PcE2F3 protein to possess two NLSs, indicating that they are a nuclear factor (Figure 2B). No NLS was predicted in PcE2F5 and 6 protein. Moreover, one leucine-rich nuclear export signal (NES) was predicted in PcE2F4 by NetNES 1.1 Server (http://www.cbs.dtu.dk/services/NetNES/, accessed on 13 July 2019). However, PcE2F1–6 proteins of large yellow croakers are all located in the nucleus using Euk-mPloc 2.0 programs (http://www.csbio.sjtu.edu.cn/bioinf/euk-multi-2/, accessed on 13 July 2019). To determine if large yellow croaker E2F1–6 were localized to the nucleus and affected by PcDP1, we performed the cherry and EGFP reporter assays. COS-7 cells were transfected with plasmid constructs expressing either a cherry-tagged E2F1–6 protein or/and an EGFP-tagged DP1 protein. As shown in Figure 4, when both DP1 and E2F1, or E2F2, or E2F3, or E2F4, or E2F5, or E2F6 co-transformed into COS-7 cells, the E2F1–6 of large yellow croakers were exclusively localized to the nucleus of the transfected cells and the DP1 proteins except for E2F4 were all enriched in the cytoplasm. DP1 was localized to the nucleus of the transfected cells in accordance with the subcellular localization of the E2F4 protein (Figure 4). Thus, E2F1–6 of large yellow croakers are the nuclear protein, respectively, and E2F4 may interact with DP1 to perform its biological function.

### 2.3. E2F1–6 Expression Pattern in Healthy Large Yellow Croaker

**Figure 5:**
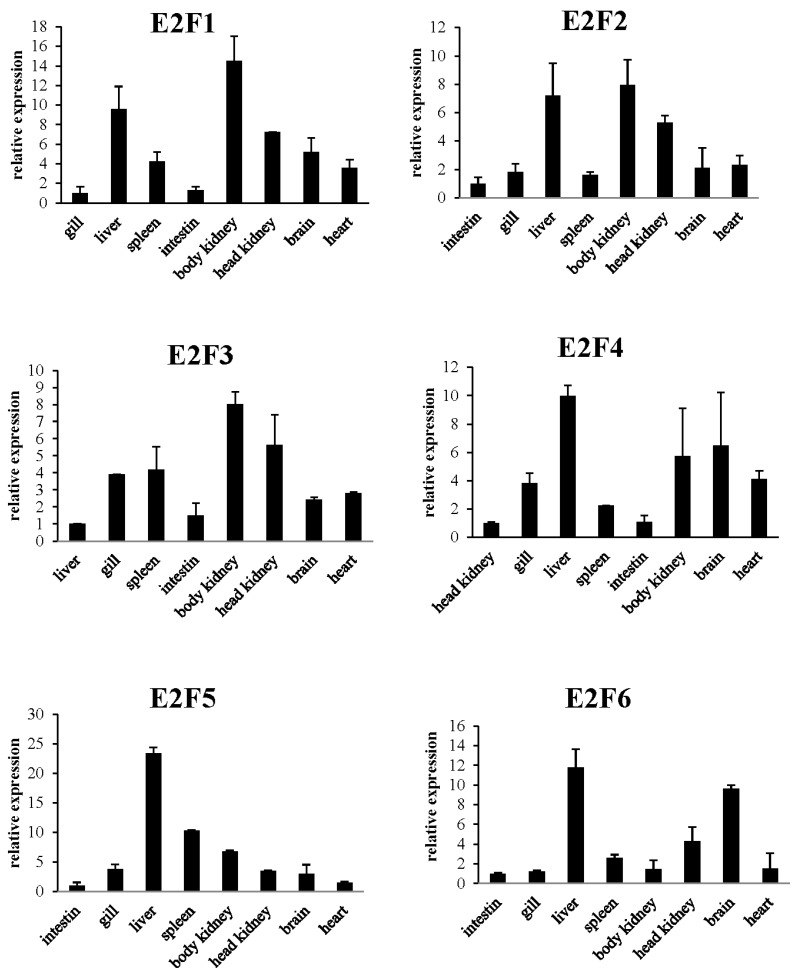
Tissue distributions of PcE2Fs mRNA in a healthy large yellow croaker. Tissues tested include the intestine, gill, liver, spleen, body kidney, head kidney, brain, and heart. Large yellow croaker *β-actin* gene expression was used as an internal control. The PcE2F1–6 mRNA level was compared with the expression level in the gill, intestine, liver and head kidney to determine the relative fold change. Vertical bars represent the mean ± SD (*n* = 3).

Primers designed, respectively, to specifically amplify the *PcE2F1*, *PcE2F2*, *PcE2F3*, *PcE2F4*, *PcE2F5*, and *PcE2F6* transcripts were used in the RT-PCR analysis of tissue distribution in healthy large yellow croakers. As shown in Figure 5, *PcE2F1–6* were expressed in all detected tissues, such as the liver, spleen, intestine, head kidney, kidney, brain, gill, and heart. Further RT-PCR analysis showed that *PcE2F1* and *PcE2F2* are highly expressed in the liver, head kidney, and kidney, and expressed lower in the spleen, intestine, brain, gill, and heart. The similarity expression pattern gives rise to the possibility that *PcE2F1* and *PcE2F2* may have overlapping or complementary functions in these organs. As the activator subclass, *PcE2F3* is also highly expressed in the head kidney and kidney, but lower in other tissues. In addition, the repressor subclass (*PcE2F4*, *PcE2F5*, and *PcE2F6*) showed differently expressed patterns in healthy large yellow croakers. Similarly, the transcriptional expression level of the three genes in the liver is relatively high, especially *PcE2F5* where the relative expression value is about 15-fold. Interestingly, we observed that *PcE2F4* and *PcE2F6* are expressed more highly in the brain than in other tissues, which may suggest that the two genes are involved in the biological process of brain development and cell cycle.

### 2.4. Evaluation of Interaction of PcE2F1–6 with DP1

The dimerization domain is the E2F and DP1 protein interaction interface. The identical residues in the dimerization domain contributes to the overall stability of the E2F-DP heterodimer [31]. Sequence alignment of PcE2F1–6 shows that they all possess one dimerization domain, and in total 11 residues are identical within the dimerization domain (Figure 2A). To determine if PE2F1–6 proteins can interact with the cytoplasm protein DP1, we performed a direct yeast two-hybrid analysis. Yeast cells expressing GAL4-BD fused DP1 (bait) were mated with cells expressing GAL-AD tagged E2F1, or 2, or 3, or 4, or 5, or 6 (preys). Growth of blue yeast cells co-expressing GAL4 BD-DP1 and GAL4 AD-E2F4 fusion proteins was readily observed on selective SD/–Ade/–His/–Leu/–Trp X-gal plates; however, no cell growth was observed on selective plates when yeast cells transformed with pGBKT7-DP1 plasmids were mated with cells expressing pGADT7-E2F1, or 2, or 3, or 5, or 6 fusion proteins (Figure 5). These results indicate that PcDP1 may be a specific interacting partner of PcE2F4 but not PcE2F1–3 and 5–6.

**Figure 6:**
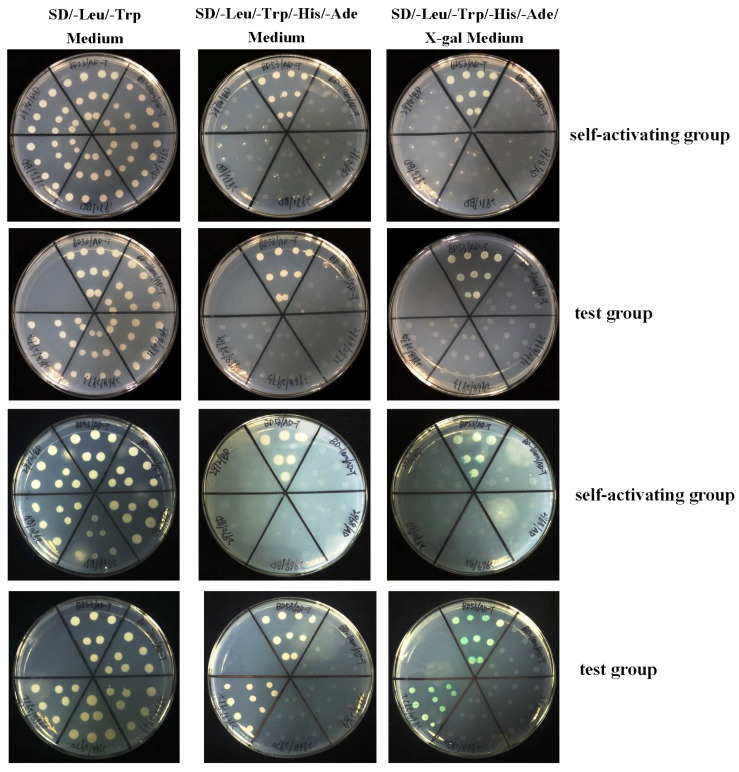
Yeast two-hybrid analysis of protein interactions between PcDP1 and PcE2Fs. Right plate: growth of yeast cells on SD/–Leu/–Trp double dropout selection medium showing successful co-transformations. Middle and left plate: growth of yeast cells on selective SD/–Ade/–His/–Leu/–Trp quadruple dropout plates (without or with X-α-gal) indicating protein–protein interactions. Notes: BD53/AD-T was positive group, BD-lam/AD-T was negative group, 2968 was DP1, 2970 was E2F1, 2971 was E2F2, 2971 was E2F3, 2972 was E2F4, 2973 was E2F5, and 2974 was E2F6.

**Figure 7:**
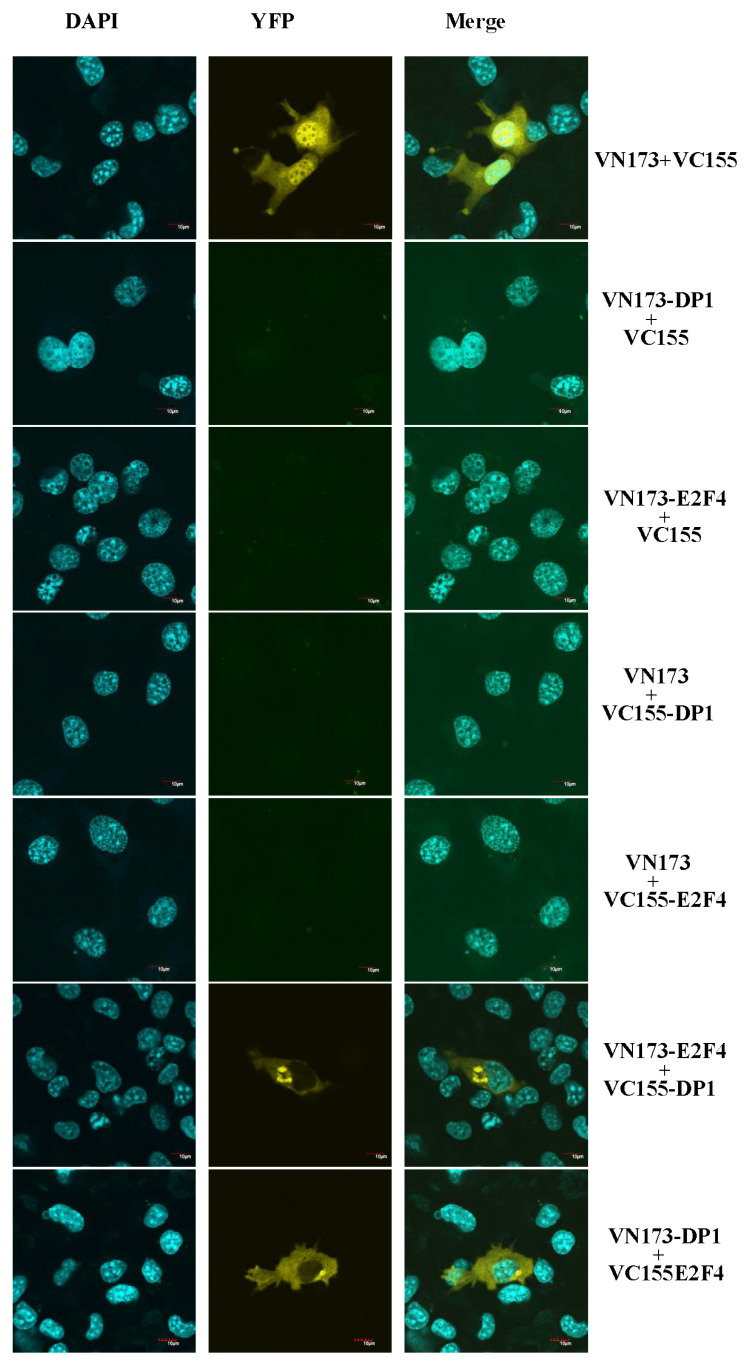
BiFC analysis of interactions between PcDP1 proteins and PcE2F4. COS-7 cells were co-transfected with equal amounts of BiFC expression constructs encoding pBiFC-VN173-PcDP1 and pBiFC-VC155-E2F4, or pBiFC-VC155-DP1 and pBiFC-VN173-E2F4 fusion proteins. Co-transfected with equal amounts of VN173 and VC155 was served as a blank control. Single transfection using the construct encoding pDP1 or pE2F4 was served as a negative control. YFP signals due to BiFC were measured by fluorescence confocal microscope. Scale bars, 10 μm.

Further confirmation of the interaction between E2F4 and DP1 was performed by bimolecular fluorescence complementation analysis. As shown in Figure 6, yellow fluorescence filled the whole cell from nucleus to cytoplasm when empty pBiFC-VN173 and pBiFC-VC155 were co-transfected into COS-7. However, yellow fluorescence signals were observed in discrete cellular regions corresponding to the nucleus with pBiFC-VN173-PcDP1 and pBiFC-VC155-E2F4, or pBiFC-VC155-DP1 and pBiFC-VN173-E2F4. In contrast, a single transfection using pBiFC-VN173-E2F4 or DP1 and pBiFC-VC155-E2F4 or DP1 induced no green fluorescence signal, serving as a negative control (Figure 7). Collectively, the results indicated that PcDP1 is a specific interacting partner of PcE2F4 and that they can form a heterodimer.

## 3. Discussion

The E2F transcription factor family is distributed widely in higher eukaryotes, including mammals, worms, fruit flies, and plants, which are very conservative in evolution [4,32,33,34]. The functional study of E2Fs has gradually attracted attention in aquatic animals. Currently, five types of E2Fs genes have been identified, namely E2F2 from *P. monodon* [35], E2F3 from *Apostichopus japonicus* [36], E2F4 and E2F5 from zebrafish and *Lethenteron reissneri* [16,37,38,39], and E2F8 from *Lethenteron reissneri* [39] and their functions were found to be related to cell and tissue proliferation and host immunity. To determine all large yellow croaker E2F genes, its genome was scrutinized for genes containing motifs homologous to the E2F DNA-binding domain and dimerization domain. Fortunately, eight genes were detected that were related to E2Fs (PcE2F1–8) in this study, but they were located on different chromosomes and possessed different structures (Figure 1). Furthermore, prediction of E2F family protein conserved domains revealed that PcE2F1–6 have one N-terminally located DNA-binding domain (DBD) and one dimerization partner binding domain (DD) following the DBD, while PcE2F7–8 only possess two duplicate DBDs containing the residues needed for DNA-binding and dimerization but cannot form complexes with DPs. This indicated that PcE2F1–6 belong to the classical E2F proteins and PcE2F7–8 to atypical group according to the E2F family taxonomy in mammals [2]. In this study, our purpose is to detect the target E2F proteins associated with a dimerization partner (PcDP1) of large yellow croakers. Therefore, we will mainly focus on the typical E2F family proteins of large yellow croakers.

Besides the DBDs and DDs, the typical E2F family proteins PcE2F1–5 in large yellow croakers, but not PcE2F6, shared a transactivation domain and a pocket protein-binding domain, and the latter sequester in dimerization domains and the transactivation domain (Figure 2). It has been confirmed that the pocket protein-binding domain can specifically interact with distinct members of the pRB family proteins (pRb, p130 and p170) to form trimeric complexes (E2F1–3 with pRB, and E2F4–5 with p107 or p130) restricting cell cycle progression and apoptosis [4]. In quiescent cells, Rb associates with E2Fs, resulting in the repression of proliferation-associated genes. As cells progress into the cell cycle, cyclin-dependent kinases (CDks) phosphorylate Rb proteins to release E2Fs, freeing E2F which can directly transactivate genes required for S-phase entry [40]. For example, the E2F-1/DP-1 heterodimer appears to be inactivated through its binding to hypophosphorylated pRb in G_1_ [41,42]. Conversely, phosphorylation of both pRb and E2F-1 [43] in late G_1_ results in the release of active E2F-1/DP-1 transcription factors and the transient expression of E2F-1-dependent genes. It is noteworthy that a linker for the activation of T cells (LAT) was identified in PcE2F5 following the dimerization domain, which plays an essential role in T cell development in mice [44]. LAT-deficient mice have a blocked thymocyte development at the immature CD25^+^ CD44^−^, CD4^−^ CD8^−^ stage and a complete lack of mature peripheral T cells [44]. However, the function of E2Fs’ LAT on T cell development have not been reported. Finally, conservative domain analysis of PcE2F6 showed that, like mammals, it lacks the C-terminal transactivation and pocket protein-binding domains, indicating that PcE2F can also act as a repressor of E2F-responsive genes by binding to promoters, preventing activation by E2F family members [45].

Accumulating evidence has suggested that E2F activity is regulated by cell cycle-dependent changes in subcellular localization [2,5,25,46]. Through searching nuclear localization signals (NLSs) on the amino acid sequence of the PcE2F1–6 protein, we found that the C-terminal of PcE2F1–4, but not E2F5–6, contains nuclear localization signals. Moreover, a nuclear export signal domain (NES) sequestering in the dimerization domain of PcE2F4 was also identified, but it was not excited in the amino acid of E2F4 in mammals. Curiously, PcE2F1–6 are all found almost exclusively in the nucleus of COS-7, which is the same with E2F1–3 nuclear localization in mammals and different from cytoplasm localization of E2F4–5 in mammals. In mammals, many studies have revealed that the subcellular localization of E2F4 and E2F5 is dependent on the cell cycle, which can be regulated through association with other factors (pocket protein or DP protein) [25,46,47]. Co-expression of p107, p130, and DP2, but not DP1, can promote nuclear localization of E2F4 and E2F5 in quiescent cells or cycling cells [47], which might be attributable to the NLS of p107, p130, and DP2 in mammals. On the other hand, Müller et al. (1997) found that the subcellular localization of the E2F-DP1 complexes is decided by the E2F [5]. They revealed that DP-1 of humans is localized in the nucleus when co-expressed with E2F1 and in the cytoplasm when co-expressed with E2F4. However, co-expression of PcDP1 and PcE2F4 can promote the transfer of PcDP1 from cytoplasm to nucleus in this study, but other PcE2Fs have no effect on subcellular localization of PcDP1 which is still distributed in cytoplasm. Thus, the subcellular localization of PcDP1 is regulated by PcE2F4, providing a potential possibility that DP1 of large yellow croakers might a partner of PcE2F4 to form the heterodimer PcE2F4/PcDP1.

Recent studies showed that E2F proteins are not only aspects controlling cell proliferation and differentiation, but that they also have tissue-specific, developmentally regulated functions [48]. Thus, the idea that E2F proteins are broadly expressed in adult tissues and display tissue-specific expression patterns to play diverse roles in different tissues has been accepted [6,49,50]). In the present study, we characterized PcE2F1–6 by evaluating their expression in different tissues of a healthy adult large yellow croaker. RT-qPCR analysis indicated that PcE2F1–6 are also constitutively expressed in all tissues tested in healthy large yellow croakers. Interestingly, higher PcE2F1–2,4–6 mRNA level were all detected in the liver of a healthy large yellow croaker. As is well known, the fish liver, as a key organ, controls many life functions and plays a prominent role in fish physiology, both in anabolism (proteins, lipids, and carbohydrates) and catabolism (nitrogen, glycogenolysis, and detoxication, etc.), as well as in vitellogenesis. Furthermore, fish hepatocytes possess strong differentiation ability [51]. All those results indicated that fish liver is a highly proliferative tissue, which might be regulated by PcE2F1–6 through cell cycle checkpoints. In addition, we found that PcE2F4 and PcE2F6 were also highly specifically expressed in the brain of large yellow croakers, which is perhaps due to the fact that PcE2F4 and PcE2F6, as a suppressor, can keep highly differentiated neurons in a quiescent phase so they no longer enter the cell cycle. This is also verified by the consistently low expression level of activators (E2F1) in the brain [52]. It is noteworthy that PcE2F1–3 were also highly specifically expressed in the kidney and PcE2F4–5 were expressed in the liver of large yellow croakers, which indicates that they might be involved in the pathways related to the immune system. This view has been supported by the expression pattern of E2F after pathogen stimulation and its regulated target genes in different tissues. For example, E2F-1-free stimulates the NF-kB signaling pathway, leading to activation of monocytes [53]. E2F2 directly regulates the STAT1 and PI3K/AKT/NF-κB pathways to exacerbate the inflammatory phenotype in rheumatoid arthritis synovial fibroblasts and mouse embryonic fibroblasts [54]. E2F3 can promote upregulation of IL-1 α, IL-β, TNF-α, and MyD88 through direct binding to their promoters [55]. Expression of E2F4 gene was upregulated in larvae Chinese oak silkworm (*Antheraea pernyi*) by bacterial (*Escherichia coli*, *Micrococcus luteus*), viral (nuclear polyhedrosis virus), and fungal (*Beauveria bassiana*) pathogens [56]. However, in zebrafish, the expression of E2F4 shows different phenomena, with lowest expression in the liver [39], which may be related to the fish species.

Heterodimerization between an E2F polypeptide and a DP polypeptide is required for the generation of a functional E2F transcription factor capable of high affinity, sequence-specific DNA binding [57,58,59]. Previous studies in our laboratory found that only DP1 has been found in the genome of large yellow croakers, and it displays ubiquitous expression in different tissues of adults [28] (. In mammals, DP1, as a heterodimerization partner, can interact with members of the E2F family (E2F1–E2F6) to increase E2F DNA-binding activity and E2F-dependent transcription in vitro [57,58,59,60]. For example, DP1 can cooperate with E2F1, E2F4, and E2F5 to induce proliferation in the epidermis of biogenic mice and serum-starved or p16INK4a-arrested cells [61,62,63,64] and with E2F1 to enhance apoptosis [65]. DP1 has also been shown in cell culture-based transformation assays to have weak oncogenic activity that is augmented by co-expression of E2F1 [66,67]. Overexpression of E2F4 in the epidermis, particularly in conjunction with DP1, results in skin tumors [61]. In this study, we found that only PcE2F4 can interact with PcDP1 by direct yeast two-hybrid and BiFC assays, indicating that the two proteins may form heterodimers to play their regulatory role. However, whether it plays the same regulatory role as the human heterodimer proteins of E2F4 and DP1 has not been reported in fish yet. Now, the dimerization domain is the PcE2F4 and PcDP1 protein interaction interface and it is believed to function in protein dimerization [68]. The crystal structure of the human E2F4-DP1 complex showed that they consist mainly of a heterodimeric coiled-coil subdomain and a heterodimeric β-sandwich subdomain that was bridged by two small helices and two small strands [31]. Furthermore, comparing the sequences of the human E2Fs, Liban et al. (2017) found that 20 residues are identical within the dimerization domain which contribute to the overall stability of the E2F-DP heterodimer and map primarily to the coiled-coil interface and the structural core that bridges the β-sandwich and coiled-coil domains [31]. In this study, we found that PcE2F4 contains one dimerization domain by prediction of the E2F family protein conserved domains (Figure 2), which also was found in PcDP1 in our previous experiment data [27]. Further analysis of the secondary structure elements of PcE2F4 and PcDP1 revealed that they both contain a varying number of helices and strands and are the similar to human E2F4 and DP1 (Figure 3). However, we noticed that there is an extra helix (α3) in the secondary structure of PcE2F4, which is also present in zebrafish E2F4 but is missing in lamprey E2F. Du et al.(2025) found that E2Fs exhibited greater diversity compared to those in jawed vertebrates [38]. In addition, there are only 11 identical residues within the dimerization domain of PcE2Fs, which is far less than the 20 identical residues within human E2Fs (Figure 2A). To understand whether these differences between larger yellow croakers and humans affects the stability of the larger yellow croaker E2F/DP1 heterodimer, and what role the extra helix (α3) plays in the formation of the PcE2F/PcDP1 heterodimer, the structural features resolution of the larger yellow croaker E2F/DP1 heterodimer may shed some light for those problem.

## 4. Materials and Methods

### 4.1. Searching E2F/DP Family Genes of Large Yellow Croaker Pseudosciaena crocea

To explore all E2F/DP family genes of large yellow croakers, the SSH cDNA library constructed in our lab and the genome of large yellow croakers from the NCBI database were used in this study. Only the genes that were homologous with E2F (Evalue ≤ 10^−5^) and contained both the DNA-binding domain and dimerization domain were considered as PcE2Fs. The functional conserved domains of E2F proteins were re-presumed using CDD software (http://www.ncbi.nlm.nih.gov/Structure/cdd/wrpsb.cgi, accessed on 16 March 2019).

### 4.2. Experimental Fish and Sampling

Healthy large yellow croakers, average body weight 150 g, were obtained from a marine cage cultured farm with 20 ± 1 °C at Xiangshan harbor, Ningbo City, Zhejiang Province, China. To amplify sequences of *E2F1*, *E2F2*, *E2F3*, *E2F4*, *E2F5*, and *E2F6* and measure its tissue distribution in healthy fish, samples including liver, spleen, intestines, kidney, head kidney, brain, gill, and heart were collected from three fish, frozen immediately in liquid nitrogen, and stored at −80 °C until use. All experiments were conducted under a protocol approved by the Medical and Laboratory Animal Ethics Sub-Committee (MLAEC, Qinzhou, China).

### 4.3. Cloning the Sequences of PcDP1 and PcE2Fs Genes of Large Yellow Croaker Pseudosciaena crocea

Total RNA was extracted from the liver, spleen, intestines, kidney, head kidney, brain, gill, and heart of healthy samples using Trizol reagent (Invitrogen, Carlsbad, CA, USA) according to the manufacturer’s instructions. The first strand cDNA was synthesized from total RNA using EasyScript One-Step gDNA Removal and cDNA Synthesis SuperMix (Transgen Biotech, Beijing, China). The specific primer for amplifying the open reading frames of PcE2F1–6 were designed based on large yellow croaker isolate SSNF genome data (https://www.ncbi.nlm.nih.gov/nuccore/1517460876, accessed on 18 March 2019) and listed in Table S1. The PCR was performed using a touchdown PCR procedure: 5 cycles of denaturation at 94 °C for 30 s, 72 °C for 3 min; 5 cycles of denaturation at 94 °C for 30 s, 70 °C for 3 s, and 72 °C for 3 min; and 25 cycles of denaturation at 94 °C for 30 s, 69 °C for 3 s, and 72 °C for 3 min. PCR products were detected on a 1.5% agarose gel and purified with a QIAquick PCR Purification Kit (Qiagen, Germantown, MD, USA). The purified PCR products were ligated into a pMD18-T Vector (TaKaRa, Osaka, Japan) and transformed into *Escherichia coli* DH5α cells. The positive clones were sequence by Thermo Fisher Scientific Co., Ltd. (Guangzhou, China).

### 4.4. Sequence and Phylogenetic Analysis

The homology searches of the sequences were analyzed using the Basic Local Alignment Search Tool (BLAST) (https://blast.ncbi.nlm.nih.gov/Blast.cgi, accessed on 20 June 2019) program, and the open reading frames (ORFs) and amino acid sequences were identified and predicted by ORF Finder (http://www.ncbi.nlm.nih.gov/projects/gorf/orfig.cgi, accessed on 20 June 2019). The functional conserved domains of Pc-DP1 were presumed using CDD (http://www.ncbi.nlm.nih.gov/Structure/cdd/wrpsb.cgi, accessed on 11 July 2019). Nuclear localization signals and leucine-rich nuclear export signals were predicted by cNLS Mapper (http://nls-mapper.iab.keio.ac.jp/cgi-bin/NLS_Mapper_form.cgi, accessed on 13 July 2019) and NetNES 1.1 Server (http://www.cbs.dtu.dk/services/NetNES/, accessed on 13 July 2019). The secondary structure elements were predicted by the Psipred 4.0 software (http://bioinf.cs.ucl.ac.uk/psipred/, accessed on 20 July 2019). Multiple sequence alignments were performed using the Clustal X 2.0 program and visualized using GeneDoc software. Phylogenetic analyses were performed using the Neighbor-Joining method in the MEGA 7.0 with 1000 bootstraps replications.

### 4.5. Tissue Distribution of PcE2Fs in Healthy Large Yellow Croakers Pseudosciaena crocea

Transcriptional tissue distributions *PcE2Fs* in healthy large yellow croakers were detected by using real-time PCR. The total RNA from tissues mentioned in Section 2.2 were extracted as templates to synthesize the first strand cDNA using the EasyScript One-Step cDNA Removal and cDNA Synthesis SuperMix (TransGen, Beijing, China). Real-time PCR was performed with the specific primers (Table S1) designed based on the ORF sequence of *PcE2Fs* on QuantStudio™ 6 Flex Real-Time PCR System, and the *β-actin* gene was used as an internal control in all of the RT-qPCR experiments. Each reaction contains 1 μL of reverse and forward primers, 10 μL of SYBR Green Mix (TransGen, Beijing, China), and 2 μL of 1:8 diluted cDNA and RNase-free water is added to a final reaction volume of 20 μL. The program was performed as follows: 1 cycle of 10 min at 95 °C, and 40 cycles of 10 s at 95 °C, 15 s at 55 °C, and 15 s at 72 °C. The relative expression level of *PcE2Fs* mRNA were calculated using the 2^−ΔΔCt^ method [69]. Each experiment was performed in triplicate.

### 4.6. Subcellular Co-Localization Analysis

The full-length ORF of *PcDP1* was PCR-amplified using gene-specific primers containing XhoI and SalI sites (Table S1) and cloned in pEGFP-N1 vector (a gift from Jichang Jian from Fisheries College of Guangdong Ocean University) to generate the recombinant expression plasmid DP1-GFP with green fluorescence. PcE2F1–6 were cloned into pmcherry-N1 between *EcoR*I and *Xho*I sites. The full-length ORF of all PcE2F1–6 were also PCR-amplified using gene-specific primers containing between *EcoR*I and *Xho*I sites (Table S1) and cloned into pmcherry-N1 vector (Wuhan Miaoling Bioscience & Technology Co. Ltd., Wuhan, China) to generate the recombinant expression plasmid E2F1-Cherry, E2F2-Cherry, E2F3-Cherry, E2F4-Cherry, E2F5-Cherry, and E2F6-Cherry with red fluorescence.

COS-7 cells were plated onto 14 mm diameter Poly-D-lysine-coated coverslips (Shanghai Solarbio Bioscience & Technology Co., Ltd., Shanghai, China) placed inside 24-well plates. Twenty-four hours after seeding, cells were co-transfected with a GFP and mCherry fusion constructs or pEGFP-N1 and pmcherry-N1empty vectors. Forty-eight hours after transfection, cells on coverslips were washed with 1 × PBS three times and fixed in paraformaldehyde for 30 min followed by DAPI staining. Images were taken by the confocal fluorescence microscope (Olympus, FV10i-Oil, Tokyo, Japan).

### 4.7. Direct Yeast Two-Hybridization

Protein interactions between PcDP1 and PcE2F1–6 were assessed by direct yeast two-hybrid analysis using the Matchmaker Two-Hybrid System (Clontech Laboratories, Mountain View, CA, USA). The coding region of PcDP1 was cloned in-frame into pGBKT7 vector to generate BD constructs (pGBKT7-DP1). The coding regions of PcE2F1–6 were cloned in-frame into the pGADT7 vector, generating AD constructs (pGADT7-E2F1-6). Each pair of pAD and pBD derivatives were transformed into *Saccharomyces cerevisiae* AH109 using the LiAc Yeast Transformation Procedure (Clontech, USA). All transformants were tested for toxicity and auto-activation before mating according to the manufacturer’s protocol. The yeast cells expressing BD-DP1 were mated with cells expressing either AD-E2F1, or 2, or 3, or 4, or 5, or 6 fusion proteins. After mating at 30 °C for 24 h, yeast cells were plated on SD/–Leu/–Trp double dropout selection medium (lacking leucine and tryptophan) plates and incubated at 30 °C for 3–5 days. Single blue colonies (>2 mm) were selected and streaked onto fresh SD/–Ade/–His/–Leu/–Trp quadruple dropout plates (lacking adenine, histidine, leucine, and tryptophan without or supplemented with X-Gal and Aureobasidin A).

### 4.8. Bimolecular Fluorescence Complementation (BiFC)

The BiFC plasmids, pBiFC-VN173 and pBiFC-VC155, were obtained from Wuhan Miaoling Bioscience & Technology Co. Ltd., China. Coding regions of PcDP1 and PcE2F4 were PCR-amplified and cloned into vectors pBiFC-VC155 and pBiFC-VN173 respectively, generating pBiFC-VN173-PcDP1, pBiFC-VC155-DP1, pBiFC-VC155-E2F4, and pBiFC-VN173-E2F4 fusion constructs. COS-7 cells were co-transfected with equal amounts of a VN and VC fusion construct. Single transfections using either pBiFC-VN173-PcDP1 or pBiFC-VN173-E2F4 were served as negative controls. YFP signals due to BiFC were measured by a confocal fluorescence microscope (Olympus, FV10i-Oil, Tokyo, Japan).

## Data Availability

Data is contained within the article or Supplementary Material.

## Supplementary Materials

The following supporting information can be downloaded at: https://www.mdpi.com/article/10.3390/ijms26115343/s1.
