# Supplementary material for: Large Yellow Croaker (Pseudosciaena crocea, Richardson) E2F4, a Cyclin-Dependent Transcription Factor, Forms a Heterodimer with DP1
Source: Int J Mol Sci. 2025 Jun 2;26(11):5343. doi: 10.3390/ijms26115343 (PMC12154468; doi:10.3390/ijms26115343)
Supplement: Supplementary file 1 [file ijms-26-05343-s001.zip › ijms-3447718-supplementary.pdf]

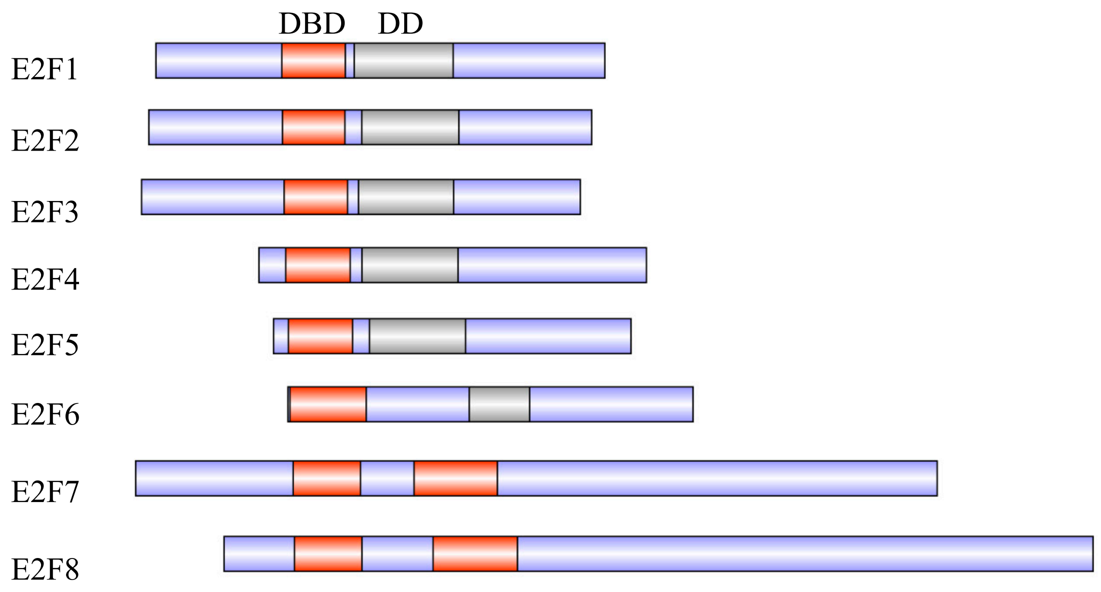

Figure S1. Schematic representation of the domain structure of full-length E2F1–8 proteins. The DNA binding and Dimerization domain are indicated by orange and gray boxes, respectively.The DNA binding domain (DBD) and Dimerization domain(DD) are indicated by orange and gray, respectively. E2F1: large yellow croaker E2F1 (NP_005216.1), E2F2: large yellow croaker E2F2 (NP_004082.1), E2F3: large yellow croaker E2F3(NP_001940.1), E2F4: large yellow E2F4 (NP_001941.2), E2F5: large yellow croaker E2F5 (NP_001942.2), E2F6: large yellow croaker E2F6 (NP_937987.2).

```
pE2F1 : MSETLITGQTSEDLLADFETLLNSGSIDLGEDHQIVIITSPSNEGLHPAA---APTSAG----EILLFATPQGPADVGIQ :  73
tE2F1 : MSETLITGQTSEDLLADFETLLNSGSINLDEDHQIVIITSPSNEGLHSAP---APTSTG----EILLFATPQGPADVGIQ :  73
zE2F1 : MSETLITGQTSEDLLADFETLLNSGSINLDEDHQIVIITSPSNEGLHPAP---APTSTG----EILLFATPQGPADVGIQ :  73
hE2F1 : --MALAGAPAGGPCAPALEALLGAGALRLLDSSQIVIISAAQDASAPPAPTGPAAPAAGPCDPDLLLFATPQAPRPT--P :  76
mE2F1 : ----MAVAPAGGQHAPALEALLGAGALRLLDSSQIVIISTAPDVGAPQLP---AAPPTGPRDSDVLLFATPQAPRPA--P :  71
bE2F1 : --MAVAGAPAGGSCAPALEALLGAGALRLLDSSQIVIISTAQDASAPPAPAGPAAPAAGPRDPDLLLFATPQAPRPT--P :  76

pE2F1 : DKRRPALGRPPVKRKLDLDSDHQYVSTTRPSIGQAPPSTPAPPRVPRTTTEKSRYDTSLNLTTKRFLNLLSQSADGVVDL : 153
tE2F1 : DKRRPPLGRPPVKRKLDLDSDHQYVSTTRPSTGQAPLSTPAPPRVPRTTAEKSRYDTSLNLTTKRFLDLLSQSADGVVDL : 153
zE2F1 : DKRRPSLGRPPVKRKLDLDSDHQYVSTTRPSTGQAPLSTPAPPRVPRTTAEKSRYDTSLNLTTKRFLDLLSQSADGVVDL : 153
hE2F1 : SAPRPALGRPPVKRRLDLETDHQYLAE---SSGPARGRGRHPGKGVKSPGEKSRYETSLNLTTKRFLELLSHSADGVVDL : 153
mE2F1 : SAPRPALGRPPVKRRLDLETDHQYLAG---SSGPFRGRGRHPGKGVKSPGEKSRYETSLNLTTKRFLELLSRSADGVVDL : 148
bE2F1 : SAPRPALGRPPVKRRLNLETDHQYLAE---SSGPARGRGRHPGKGVKSPGEKSRYETSLNLTTKRFLELLSRSADGVVDL : 153
```

DNA Binding domain **Dimerization domain**

```
pE2F1 : NWASQVLDVQKRRIYDITNVLEGIQLISKKSKNNIQWLGNRVDTALVSRHKELQREVCDLTEAEEQLDELISKCNLQLRL : 233
tE2F1 : NWASQVLDVQKRRIYDITNVLEGIQLISKKSKNNIQWLGNRIDAALVSRHKELQREVCDLTDAEEQLDDLISKCNLQLRL : 233
zE2F1 : NWASQVLDVQKRRIYDITNVLEGIQLISKKSKNNIQWLGNRIDAALVSRHKELQREVCDLTDAEEQLDELISKCNLQLRL : 233
hE2F1 : NWAAEVLKVQKRRIYDITNVLEGIQLIAKKSKNHIQWLGSHTTVGVGGRLEGLTQDLRQLQESEQQLDHLMNICTTQLRL : 233
mE2F1 : NWAAEVLKVQKRRIYDITNVLEGIQLIAKKSKNHIQWLGSHTMVGIGKRLEGLTQDLQQLQESEQQLDHLMHICTTQLQL : 228
bE2F1 : NWAAEVLKVQKRRIYDITNVLEGIHLIAKKSKNHIQWLGSHATVGISGRLEGLTQDLQQLQESEQQLDHLLHTCSTQLRL : 233
```

Dimerization domain

```
pE2F1 : LTEDPQNKKLGYVRCQDLRKSFDSPDQLVMVIRAPPETQMQVSEPSKGYQVSLKSTRGPIDVFLCPDDSSSVCSPVTGSS : 313
tE2F1 : LTEDPQNKKLGYVRCQDLRQSFDSPDQLVMVIRAPPETQMQVSEPSEGYQVSLKSTRGPIDVFLCPEDSSGVCSPVTGGS : 313
zE2F1 : LTEDPQNKKLGYVRCQDLRQSFDSPDQLVMVIRAPPETQMQVSEPSEGYQVSLKSTRGPIDVFLCPEDSSGVCSPVTGGS : 313
hE2F1 : LSEDTDSQRLAYVTCQDLRSIADPAEQMVMVIKAPPETQLQAVDSSENFQISLKSKQGPIDVFLCPEET------VGGIS : 307
mE2F1 : LSEDSDTQRLAYVTCQDLRSIADPAEQMVIVIKAPPETQLQAVDSSETFQISLKSKQGPIDVFLCPEES------ADGIS : 302
bE2F1 : LSEDADSQRLAYVTCQDLRSIADPAEQMVMVIKAPPETQLQAVDSSENFQISLKSKQGPIDVFLCPEES------VGGTS : 307

pE2F1 : PSKPNADPPMVPPPAQPAEQSQARTSTAALEVGLSSPASTSSTVTAASQQDPSSLVLGGDTESLLGGDPFASLGDMPDFD : 393
tE2F1 : PSKPNADPSLVQPPTQPTDQSQPRTCSTALEVGLSSPASTSSTVTAVSQQDPSSLVLGGDTESILGGDPFSNLGDLPDFD : 393
zE2F1 : PSKPIADPSLVQPSTQPTDQSQPRTCSTALEVGLSSPASTSSTVTAVSQQDPSSLVLGGDTESILGGDPFSNLGDLPDFD : 393
hE2F1 : PGK---------TPSQEVTSEEENRATDSATIVSPPPSSPPSSL----TTDPSQSLLSLEQEPLLSR--MGSLRAPVDED : 372
mE2F1 : PGK---------TSCQETSSGE-DRTADSGPAG-PPPSPPSTSP----ALDPSQSLLGLEQEAVLPR--MGHLRVPMEED : 365
bE2F1 : PGK---------TPSQGAASGEEDRTADLATAVPPPPSSPRSSP----ATDPSQSLLSLEQEPLLSR--MGGLRAPVDED : 372

pE2F1 : -LSPLSSSD----FLNGEGLSLPLDGFINLSPPHS-HDYHFGLEDHEGISELFDCDFGDLSQVLGDS : 454
tE2F1 : -LSPLSSTD----FLNVEGLPLPLDGFINLSPPHS-HDYHYGLEDHEGISELFDCDFGDLSQVLGAD : 454
zE2F1 : -LSPLSSTD----FLNGEGLPLPLDGFINLSPPHS-HDYHYGLEDHEGISELFDCDFGDLSQVLGDD : 454
hE2F1 : RLSPLVAADSLLEHVREDFSGLLPEEFISLSPPHEALDYHFGLEEGEGIRDLFDCDFGDLTPLDF-- : 437
mE2F1 : QLSPLVAADSLLEHVKEDFSGLLPGEFISLSPPHEALDYHFGLEEGEGIRDLFDCDFGDLTPLDF-- : 430
bE2F1 : RLSPLVAADSLLEHVKEDFSSLLPEEFITLSPPHEALDYHFGLEEGEGIRDLFDCDFGDLTPLDF-- : 437
```

Figure S2. Multiple sequence alignment of E2F1 proteins. Sequence alignment was performed using ClustalX 2.0 and GeneDoc software. The functional domains were determined by searching the CDD software (http://www.ncbi.nlm.nih.gov/Structure/cdd/wrpsb.cgi, accessed on 11 July 2019). The DNA binding and Dimerization domains are indicated by green and red boxes, respectively. tE2F1: tilapia E2F1 (XP_003449170.1), zE2F1: zebrafish E2F1 (XP_004574998.1), hE2F1: human E2F1 (NP_005216.1), mE2F1: mouse E2F1 (NP_031917.1), bE2F1: bovine E2F1 (NP_001193008.1).

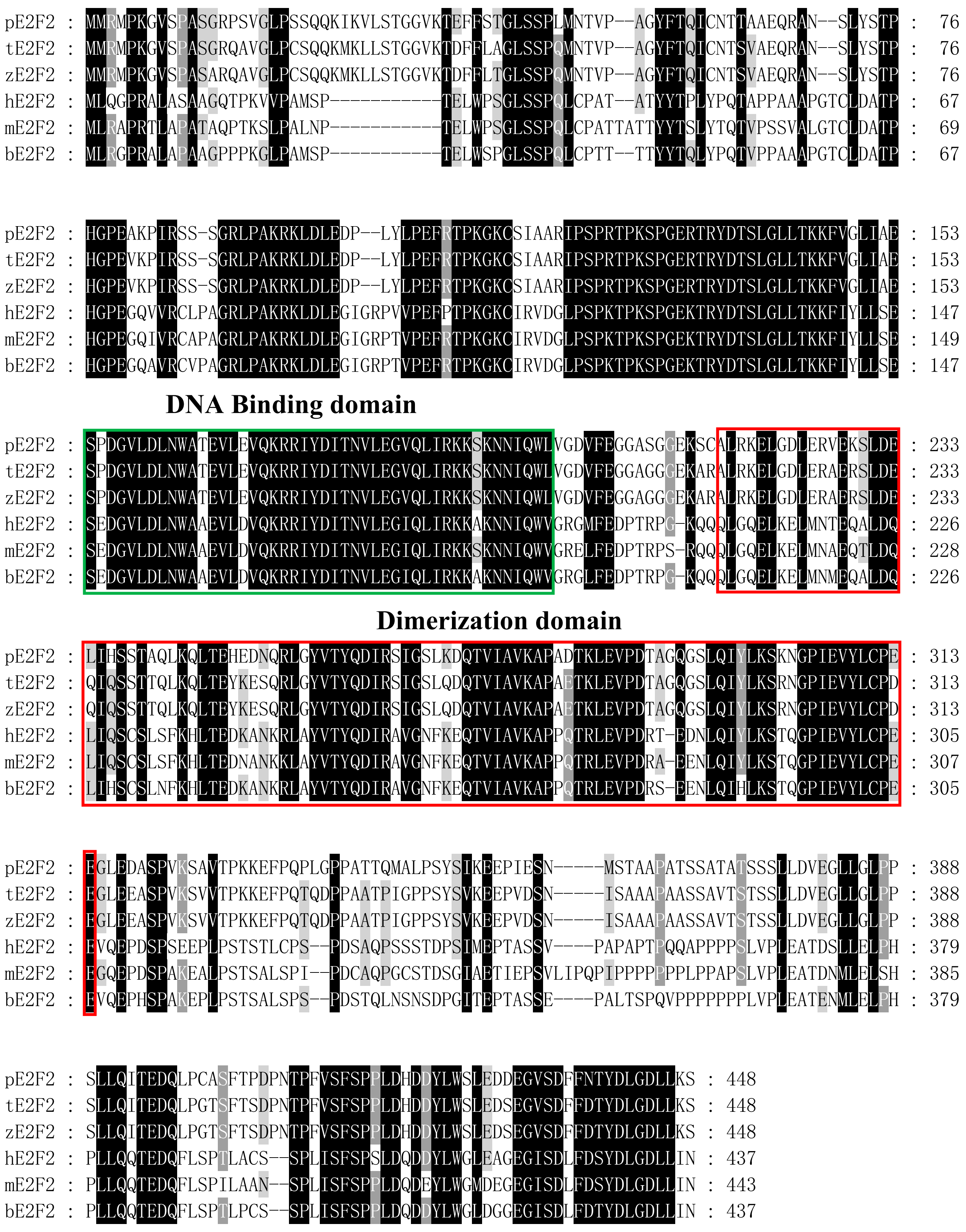

Figure S3. Multiple sequence alignment of E2F2 proteins. Sequence alignment was performed using ClustalX 2.0 and GeneDoc software. The functional domains were determined by searching the CDD software (http://www.ncbi.nlm.nih.gov/Structure/cdd/wrpsb.cgi, accessed on 11 July 2019). The DNA binding and Dimerization domains are indicated by green and red boxes, respectively. tE2F2: tilapia E2F2 (XP_005477217.1), zE2F2: zebrafish E2F2 (XP_004540481.1), hE2F2: human E2F2 (AAX42851.1), mE2F2: mouse E2F2 (EDL29946.1), bE2F2: bovine E2F2 (XP_024838443.1).

```
pE2F3 : MRRGISSAPDKVFLAGVGG------------SPLDNNIILTTLSDR--LNPGQSNATFIQIITTPP---PCNVTQTS :  60
tE2F3 : MRRGTSSAQEKVILAGVGG------------SSLDNNIILTALSDR--LNPGQSNATYIQIITTPP---PCNVTQTS :  60
zE2F3 : MRRGTSSAQEKVILAGVGG------------SSLDNNIILTALSDR--LNPGQSNATYIQIITTPP---PCNITQTS :  60
hE2F3 : MRKGIQPALEQYLVTAGGGEGAAVVAAAAAASMDKRALLASPGFAAAAAAAAAPGAYIQILTTNTSTTSCSSSLQS :  76
mE2F3 : MRKGIQPALEQYLVTAGGGEGAAVVAAAAAASMDKRALLASPGFA----AAAAPGTYIQILTTNPSTTSCATSLQS :  72
bE2F3 : MRKGIQPALEQYLVTAGGGEGAAVVAAAAAASMDKRALLASPGFP--AAAAAAPSAYIQILTTNTSTTSCSSSLQS :  74

pE2F3 : NVCLSEPQIN-----------NIYTTPQQAAANG--AGQRPALGR--------PPAKRRLALDDSDHQYQSEPTRT : 115
tE2F3 : NVCLSEPQIN-----------NIYTTPQQAAANA--AGQRPALGR--------PPAKRRLALDDSDHQYQTEPAKT : 115
zE2F3 : NVCLSEPQIN-----------NIYTTPQQAAANA--AGQRPALGR--------PPAKRRLALDDSDHQYQTEPAKT : 115
hE2F3 : GAVAAGPLLPSAPGAEQTAGSLLYTTPHGPSSRAGLLQQPPALGRGGSGGGGGPPAKRRLELGESGHQYLSDGLKT : 152
mE2F3 : GALTAGPLLPSVPGTEP-AASSLYTTPQGPSSRVGLLQQPPAPGRGG---GGGPPAKRRLELGESGHQYLSDGLKT : 144
bE2F3 : GAVAAGPLLPSAPGVEQTAGSLIYTTPHGPSGRAGLLQQPPALGRGGSGGGGGPPAKRRLELGESGQQYLSDGLKT : 150
```

DNA Binding domain

```
pE2F3 : PRGRGGTASANGARLKTPRTPKSPPEKTRYDTSLGLLTKKFVDLLAQSSDGVLDLNLAAETLQVQKRRLYDITNVL : 191
tE2F3 : PRGRGGAAASNGARLKTPRTPKSPPEKTRYDTSLGLLTKKFVDLLAQSSDGVLDLNLAAETLQVQKRRLYDITNVL : 191
zE2F3 : PRGRGGAAASNGARLKTPRTPKSPPEKTRYDTSLGLLTKKFVDLLAQSSDGVLDLNLAAETLQVQKRRLYDITNVL : 191
hE2F3 : PKGKGRAALRS---PDSPKTPKSPSEKTRYDTSLGLLTKKFIQLLSQSPDGVLDLNKAAEVLKVQKRRIYDITNVL : 225
mE2F3 : PKGKGRAALRS---PDSPKTPKSPSEKTRYDTSLGLLTKKFIQLLSQSPDGVLDLNKAAEVLKVQKRRIYDITNVL : 217
bE2F3 : PKGKGRAALRS---PDSPKTPKSPSEKTRYDTSLGLLTKKFIQLLSQSPDGVLDLNKAAEVLKVQKRRIYDITNVL : 223
```

DNA Binding domain **Dimerization domain**

```
pE2F3 : EGIHLIKKKSKNNIQWMGCSLLEVEGALSQRQRLTAEVSALGEEEQRLEQLIQRCSLDMRHMSELPSNQKYAYVVTY : 267
tE2F3 : EGIHLIKKKSKNNIQWMGCSLLEVEGALSQRQRLTAEVSALADEEQRLEQLIQRCSLDMRHMSELPGNQKYAYVVTY : 267
zE2F3 : EGIHLIKKKSKNNIQWMGCSLLEVEGALSQRQRLTAEVSALADEEQRLEQLIQRCSLDMRHMSELPGNQKYAYVVTY : 267
hE2F3 : EGIHLIKKKSKNNVQWMGCSLSEDGGMLAQCQGLSKEVTELSQEEKKLDELIQSCTLDLKLLTEDSENQRLAYVVTY : 301
mE2F3 : EGIHLIKKKSKNNVQWMGCSLSEDGGMLAQCQGLSKEVTELSQEEKKLDELIQSCTLDLKLLTEDSENQRLAYVVTY : 293
bE2F3 : EGIHLIKKKSKNNVQWMGCSLSEDGGMLAQCQGLSKEVTELSQEEKKLDELIQSCTLDLKLLTEDSENQRLAYVVTY : 299
```

Dimerization domain

```
pE2F3 : QDIKQLGNLKDQTVIVVKAPTDTKLEVPDPDESLSIHLTSTKGPIEVLLCPDEENDPRSPVKNGNMDINGNSPFLK : 343
tE2F3 : QDIKQ-GSLRDQTVIVVKAPTDTKLEVPDPEESLSIHLTSTKGPIEVLLCPDEENDPRSPVKNGNTDINGNSPFLK : 342
zE2F3 : QDIKQ-GSLRDQTVIVVKAPTDTKLEVPDPEESLSIHLTSTKGPIEVLLCPDEENDPRSPVKNGNTDINGNSPFLK : 342
hE2F3 : QDIRKISGLKDQTVIVVKAPPETRLEVPDSIESLQIHLASTQGPIEVYLCP-EETETHSPMKTNNQDHNGN----- : 371
mE2F3 : QDIRKISGLKDQTVIVVKAPPETRLEVPDSIESLQIHLASTQGPIEVYLCP-EETETHRPMKTNNQDHNGN----- : 363
bE2F3 : QDIRKISGLKDQTVIVVKAPPETRLEVPDPIESLQIHLASTQGPIEVYLCP-EETETHSPMKTNNQDHNGN----- : 369

pE2F3 : VLQDPSCTNTSPSPSLAPPSSSS-AVSVTTLSPISSPYTSLLQQTEDQIPSSLG-PFLNLGPPLLDQ-DDYLLGLA : 416
tE2F3 : VLQDPSGTTTSPNPFLAPPPSSS-AVSVTTLSPISSPYTSLLQQTEDQIPSSLG-PFLNLGPTLLDQEDDYLLGLG : 416
zE2F3 : VLQDPSCTTTSPNPFLAPPPSSSSAVSVTTLSPISSTYTSLLQQTEDQIPSSLG-PFLNLGPTLLDQEDDYLLGLG : 417
hE2F3 : -IPKPASKDLASTN----SGHSDCSVSMGNLSPLASP-ANLLQQTEDQIPSNLEGPFVNLLPPLLQE--DYLLSLG : 439
mE2F3 : -IPKPTSKDLASNN----SGHSDCSVSTANLSPLASP-ANLLQQTEDQIPSNLEGPFVNLLPPLLQE--DYLLSLG : 431
bE2F3 : -IPKPTSKDLASTN----SGHSDCSISMANLSPLASP-ANLLQQTEDQIPSNLEGPFVNLLPPLLQE--DYLLSLG : 437

pE2F3 : DDQGISDLFDACDFDKMPTLGLDDLLCS : 444
tE2F3 : DDQGISDLFDSCDFDKMPSLGLDELLGS : 444
zE2F3 : DDQGISDLFESCDFDKMPSLGLDELLGS : 445
hE2F3 : EEEGISDLFDAYDLEKLP--LVEDFMCS : 465
mE2F3 : EEEGISDLFDAYDLEKLP--LVEDFMCS : 457
bE2F3 : EEEGISDLFDAYDLEKLP--LVEDFMCS : 463
```

Figure S4. Multiple sequence alignment of E2F3 proteins. Sequence alignment was performed using ClustalX 2.0 and GeneDoc software. The functional domains were determined by searching the CDD software (http://www.ncbi.nlm.nih.gov/Structure/cdd/wrpsb.cgi, accessed on 11 July 2019). The DNA binding and Dimerization domains are indicated by green and red boxes, respectively. tE2F3: tilapia E2F3 (XP_003443756.11), zE2F3: zebrafish E2F3 (XP_004563728.1), hE2F3: human E2F3 (NP_001940.1), mE2F3: mouse E2F3 (NP_034223.1), bE2F3: bovine E2F3 (NP_001179767.1).

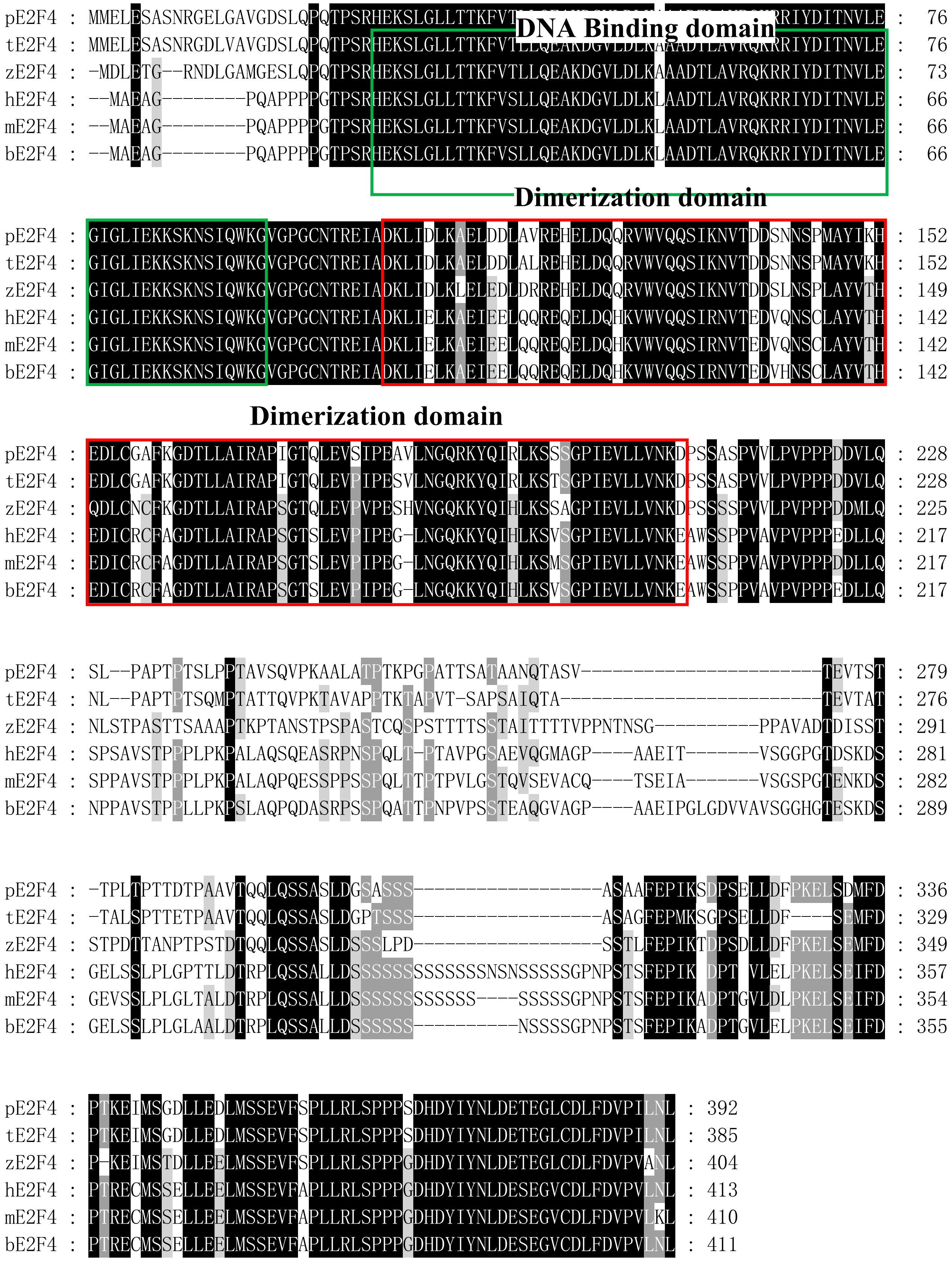

Figure S5. Multiple sequence alignment of E2F4 proteins. Sequence alignment was performed using ClustalX 2.0 and GeneDoc software. The functional domains were determined by searching the CDD software (http://www.ncbi.nlm.nih.gov/Structure/cdd/wrpsb.cgi, accessed on 11 July 2019). The DNA binding and Dimerization domains are indicated by green and red boxes, respectively. tE2F4: tilapia E2F4 (XP_003447142.1), zE2F4: zebrafish E2F4 (NP_998597.2), hE2F4: human E2F4 (NP_001941.2), mE2F4: mouse E2F4 (NP_683754.1), bE2F4: bovine E2F4 (XP_005218671.2).

```
pE2F5 : -MEFETTETVRSTP----------------------------------SRHEKSLGLLTMKFVSLLQEAKDGVLDL :  41
tE2F5 : -MEFETTETARSTP----------------------------------SRHEKSLGLLTIKFVSLLQEAKDGVLDL :  41
zE2F5 : MAESNSASFPHSTPN-------------------------------GSSRHEKSLGLLTVKFVTLLQEAKDGVLDL :  45
hE2F5 : MAAAEPASSGQQAPAGQGQGQRPPPQPPQAQAPQPPPPPQLGGAGGGSSRHEKSLGLLTTKFVSLLQEAKDGVLDL :  76
mE2F5 : MAAAEPTSSAQPTPQAQAQ---PPPHG----APSSQPSAALA---GGSSRHEKSLGLLTTKFVSLLQEAQDGVLDL :  66
bE2F5 : MAAAEPASCAPPG--------------------------------AGSSRHEKSLGLLTAKFVSLLQEAKDGVLDL :  44
```

DNA Binding domain

```
pE2F5 : KVAADSLAVKQKRRIYDITNVLEGVGLIEKKNKNIIQWRGENSGSQTQEVVEQVKVLKAQIAELEAQEKELDSQKA : 117
tE2F5 : KVAADSLAVKQKRRIYDITNVLEGVGLIEKKNKNVIQWRGENIASQTEEVLEQVNVLKAQIAELEAQEKELDNQKA : 117
zE2F5 : KVAADSLAVKQKRRIYDITNVLEGIGLIEKKTKNTIQWKGESTGCQPQEVLEQVELLKANIADLELQERELDMQKA : 121
hE2F5 : KAAADTLAVRQKRRIYDITNVLEGIDLIEKKSKNSIQWKGVGAGCNTKEVIDRLRYLKAEIEDLELKERELDQQKL : 152
mE2F5 : KAAADTLAVRQKRRIYDITNVLEGIDLIEKKSKNSIQWKGVGAGCNTKEVIDRLRCLKAEIEDLELKERELDQQKL : 142
bE2F5 : KAAADTLAVRQKRRIYDITNVLEGIDLIEKKSKNSIQWKGVGAGCNTKEVIDRLKYLKAEIEDLELKERELDQQKL : 120
```

Dimerization domain

```
pE2F5 : WLDENIKHLKHDPIVSPYKFVTHEDICNAFSGDTLLAVVAPAGTQLEVPLPEMGQSGQKKYQVNLRSHSAPIQVML : 193
tE2F5 : WLEENIKHLNHDPVLNTYKFVTHEDICSAFSGETLLAVVAPAGTQLEVPLPEMGQSGQKKYQVNLRSHSAPIQVVL : 193
zE2F5 : CLQQSIKQLNEDPYSCRYSYVMHEDICDAFSGDTLLAVMAPSGTQLEVPVPEMGHNGQKKYQVNLRSHSAPIQVML : 197
hE2F5 : WLQQSIKNVMDDSINNRFSYVTHEDICNCFNGDTLLAIQAPSGTQLEVPIPEMGQNGQKKYQINLKSHSGPIHVLL : 228
mE2F5 : WLQQSIKNVMEDSINNRFSYVTHEDICNCFHGDTLLAIQAPSGTQLEVPIPEMGQNGQKKYQINLKSHSGPIHVLL : 218
bE2F5 : WLQQSIKNVMDDSINNRFSYVTHEDICNCFNGDTLLAIQAPSGTQLEVPIPEMGQNGQKKYQINLKSHSGPIHVLL : 196
```

```
pE2F5 : INRDSDSTIPVVFSVPPTDDIGPLPTPPSTPASLQRFPLSTSVYSTSNTTSSYCSQDSLCSDHQMVLPEHNEVLTP : 269
tE2F5 : INRDSDSRVPVVFSVPPTDDICQMPTPPSTPASLQRFPLSVSTSNTN--TTSCCSQESLCSDHQMVLPEHDDVLTP : 267
zE2F5 : INRETSCSKPVVVSVPPIDDISSMPTPPSTPAGLQRFPISS---------IDLCDQK----HGLLKSPAAEHQLTP : 260
hE2F5 : INKESSSSKPVVFPVPPPDDLTQPSSQSLTPVTPQKSSMATQN-----------------------LPEQ------ : 275
mE2F5 : INKESSSSKPVVFPVPPPDDLTQPSSQSSTSVTPQKSTMAAQN-----------------------LPEQ------ : 265
bE2F5 : INKESSSSKPVVFPVPPPDDLAQPPSQPPTPVPPHKPSSAVQS-----------------------LPEP------ : 243
```

```
pE2F5 : SSTPPNLHMECHS-LSAAELEQQQMDLVGSEFQSVLDVSSLLKLNTAGDHMKDDREGTVDLIDELMSTD------- : 337
tE2F5 : SATPPDVQMVCHG-RPVMGLEQQEMDLVDQEFQSVLDVSSLLKLSATEDHMKDNQEGAVDLIDELMSTD------- : 335
zE2F5 : SSTSPDVHMECNPESPASQCLLMQSSLGGPEEQ--QRELGGQDLQSMLEEMRDEREGVSNLIDELMSSDVFPLLRL : 334
hE2F5 : ---------------HVSERSQALQQTSATDISSAGSISG-------------------DIIDELMSSDVFPLLRL : 317
mE2F5 : ---------------HVSERSQTFQQTPAAEVSS-GSISG-------------------DIIDELMSSDVFPLLRL : 306
bE2F5 : ---------------PVSERSQPLQHTPATDLSSAGSISG-------------------DIIDELMSSDVFPLLRL : 285
```

```
pE2F5 : ----GIDYSFNLDDHEGVCDLFDVQILNY------------------------- : 362
tE2F5 : ----GIDYSFNLDDNEGVCDLFDVQILNYGDDYNFNLDDNEGVCDLFDVQILNY : 385
zE2F5 : SPNPGVDYSFNLDDNEGVCDLFDVQILNY------------------------- : 363
hE2F5 : SPTPADDYNFNLDDNEGVCDLFDVQILNY------------------------- : 346
mE2F5 : SPTPADDYNFNLDDNEGVCDLFDVQILNY------------------------- : 335
bE2F5 : SPTPADDYNFNLDDNEGVCDLFDVQILNY------------------------- : 314
```

Figure S6. Multiple sequence alignment of E2F5 proteins. Sequence alignment was performed using ClustalX 2.0 and GeneDoc software. The functional domains were determined by searching the CDD software (http://www.ncbi.nlm.nih.gov/Structure/cdd/wrpsb.cgi, accessed on 11 July 2019). The DNA binding and Dimerization domains are indicated by green and red boxes, respectively. tE2F5: tilapia E2F5 (XP_005476633.1), zE2F5: zebrafish E2F5 (NP_001184229.1), hE2F5: human E2F5 (NP_001942.2), mE2F5: mouse E2F5 (NP_031918.2), bE2F5: bovine E2F5 (XP_005215763.1).

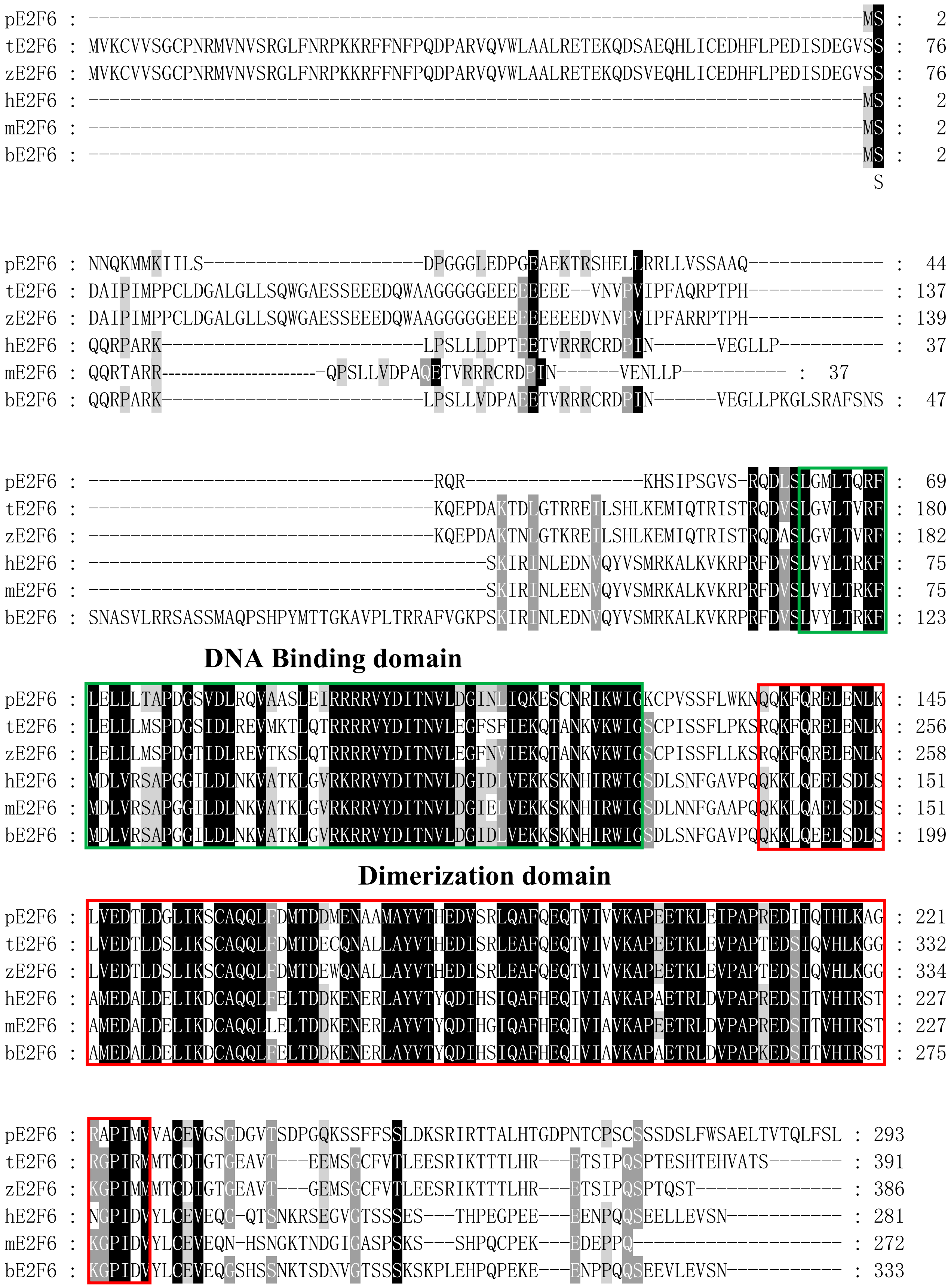

Figure S7. Multiple sequence alignment of E2F6 proteins. Sequence alignment was performed using ClustalX 2.0 and GeneDoc software. The functional domains were determined by searching the CDD software (http://www.ncbi.nlm.nih.gov/Structure/cdd/wrpsb.cgi, accessed on 11 July 2019). The DNA binding and Dimerization domains are indicated by green and red boxes, respectively. tE2F6: tilapia E2F6 (XP_019201872.1), zE2F6: zebrafish E2F6 (XP_004568502.1), hE2F6: human E2F6(CAD37950.1), mE2F6: mouse E2F6 (NP_150373.2), bE2F6: bovine E2F6 (XP_024854335.1).

Table S1. Primers used in this study.

Primer name	Sequence	Application
pMD18-DP1F	ATGGCTAAAGATGCTGGTCTGA	ORF cloning
pMD18-DP1R	TTAGTCTTCATCGTCGTTCTCGTCGT	ORF cloning
pMD18-E2F1F	ATGTCAGAGACTCTGATAACAGGGC	ORF cloning
pMD18-E2F1R	CTAACTGTCCCCCAAAACTTGTG	ORF cloning
pMD18-E2F2F	ATGATGCGGATGCCTAAAGGCG	ORF cloning
pMD18-E2F2R	TCAGCTCTTCAGCAGATCCCCAAG	ORF cloning
pMD18-E2F3F	ATGAGAAGAGGGATCTCCTCG	ORF cloning
pMD18-E2F3R	CTAGCTGCACAGGAGATCATC	ORF cloning
pMD18-E2F4F	ATGATGGAGCTGGAGTCGGCCAG	ORF cloning
pMD18-E2F4R	TCAGAGGTTGAGAATGGGGACGTC	ORF cloning
pMD18-E2F5F	ATGGAGTTTGAGACGACGGAG	ORF cloning
pMD18-E2F5R	TTAGTAATTGAGGATCTGCAC	ORF cloning
pMD18-E2F6F	ATGTCCAATAATCAGAAGATG	ORF cloning
pMD18-E2F6R	CTACAGGCTGAATAACTGCGTCA	ORF cloning
RT-DP1F	CTCATACTGCAGCAAATAGCTT	Real-time PCR
RT-DP1R	CTTCAGCACCTCGATGTCA	Real-time PCR
RT-E2F1F	TCCCAGAGTTCCTCGGACCAC	Real-time PCR
RT-E2F1R	TGTCAACTCGATTGCCAAGCC	Real-time PCR
RT-E2F2F	CAAACAACTCACTGAACACGAA	Real-time PCR
RT-E2F2R	CACTTTTAACCGGACTAGCATC	Real-time PCR
RT-E2F3F	GTATGACATCACCAATGTGCT	Real-time PCR
RT-E2F3R	TACGCATATTTCTGGTTGCTT	Real-time PCR
RT-E2F4F	ACGAGGCATCTACGATATCACT	Real-time PCR
RT-E2F4R	GACAGCCAGGTCATCTAGCTC	Real-time PCR
RT-E2F5F	GAGACTGTACGCTCGACACC	Real-time PCR
RT-E2F5R	CCACTCCTTCAAGCACGTT	Real-time PCR
RT-E2F6F	AGTGTATGACATCACCAACGTCC	Real-time PCR
RT-E2F6R	TCCTCCACCAGCTTCAGGTTC	Real-time PCR
RT-β -actinF	GATGTGGATCAGCAAGCAGG	Real-time PCR
RT-β -actinR	GAGCTGAAGTTGTTGGGTGT	Real-time PCR
EGFP-Dp1F	CCGACGTCGACATGGCTAAAGATGCTGGTCTGATTGAAAC	Co-localization
EGFP-Dp1R	CG GGATCC GTCTTCATCGTCGTTCTCGTCGTAC	Co-localization
Cherry-E2F1F	CCGCTCGAG ATGTCAGAGACTCTGATAACAGGGCAGACATC	Co-localization
Cherry-E2F1R	CGGAATTC ACTGTCCCCCAAAACTTGTGATAGGTCAC	Co-localization
Cherry-E2F2F	CCGCTCGAG ATGATGCGGATGCCTAAAGGCGTCTCTCCGGC	Co-localization
Cherry-E2F2R	CGGAATTC GCTCTTCAGCAGATCCCCAAGATCATATG	Co-localization
Cherry-E2F3F	CCGCTCGAG ATGAGAAGAGGGATCTCCTCG	Co-localization
Cherry-E2F3R	CGGAATTC GCTGCACAGGAGATCATC	Co-localization
Cherry-E2F4F	CCGCTCGAGTGATGGAGCTGGAGTCGGCCAGTAATAGAGGC	Co-localization
Cherry-E2F4R	CGGAATTC GAGGTTGAGAATGGGGACGTCAAAGAGGTC	Co-localization
Cherry-E2F5F	CCGCTCGAG ATGGAGTTTGAGACGACGGAG	Co-localization
Cherry-E2F5R	CGGAATTC GTAATTGAGGATCTGCAC	Co-localization
Cherry-E2F6F	CCGCTCGAG ATGTCCAATAATCAGAAGATGATGAAG3	Co-localization
Cherry-E2F6R	CGGAATTC CAGGCTGAATAACTGCGTCACTG3	Co-localization
BiFC-DP1F	CCGACGTCGACATGGCTAAAGATGCTGGTCTGATTGAAAC	BiFC
BiFC-DP1R	CGGGGTACC GTCTTCATCGTCGTTCTCGTCGTAC	BiFC
BiFC-E2F1F	CGGAATTCATGTCAGAGACTCTGATAACAGGGCAGACATC	BiFC
BiFC-E2F1R	TGCTCTAGAACTGTCCCCCAAAACTTGTGATAGGTCAC	BiFC
BiFC-E2F2F	CGGAATTCATGATGCGGATGCCTAAAGGCGTCTCTC	BiFC
BiFC-E2F2R	TGCTCTAGAGCTCTTCAGCAGATCCCCAAGATCATATG	BiFC
BiFC-E2F3F	CGGAATTCATGAGAAGAGGGATCTCCTCG	BiFC
BiFC-E2F3R	TGCTCTAGAGCTGCACAGGAGATCATC	BiFC
BiFC-E2F4F	CGGAATTCATGATGGAGCTGGAGTCGGCCAGTAATAGAGGC	BiFC
BiFC-E2F4R	TGCTCTAGAGAGGTTGAGAATGGGGACGTCAAAGAGGTC	BiFC
BiFC-E2F5F	CGGAATTCATGGAGTTTGAGACGACGGAG	BiFC
BiFC-E2F5R	TGCTCTAGAGTAATTGAGGATCTGCAC	BiFC
BiFC-E2F6F	CGGAATTCATGTCCAATAATCAGAAGATG	BiFC
BiFC-E2F6R	TGCTCTAGACAGGCTGAATAACTGCGTCACTG	BiFC
BD-DP1F	CGGGATCCgtATGGCTAAAGATGCTGGTCTGATTGAAAC	Yeast two-hybrid
BD-DP1R	CCGACGTCGACTTAGTCTTCATCGTCGTTCTCGTCGTAC	Yeast two-hybrid
AD-E2F1F	CGGAATTCATGTCAGAGACTCTGATAACAGGGCAGACATC	Yeast two-hybrid
AD-E2F1R	CGCTCGAGCTAACTGTCCCCCAAAACTTGTGATAGGTCAC	Yeast two-hybrid
AD-E2F2F	CGGAATTCATGATGCGGATGCCTAAAGGCGTCTCTCCGGC	Yeast two-hybrid
AD-E2F2R	CCGCTCGAGTCAGCTCTTCAGCAGATCCCCAAGATCATATG	Yeast two-hybrid
AD-E2F3F	CGGAATTCATGAGAAGAGGGATCTCCTCG	Yeast two-hybrid
AD-E2F3R	CCGCTCGAGCTAGCTGCACAGGAGATCATC	Yeast two-hybrid
AD-E2F4F	CGGAATTCATGATGGAGCTGGAGTCGGCCAGTAATAGAGGC	Yeast two-hybrid
AD-E2F4R	CCGCTCGAGTCAGAGGTTGAGAATGGGGACGTCAAAGAG	Yeast two-hybrid
AD-E2F6F	CGGAATTCATGTCCAATAATCAGAAGATG	Yeast two-hybrid
AD-E2F6R	CCGCTCGAGCTACAGGCTGAATAACTGCGTCA	Yeast two-hybrid

Notes: Red indicates enzyme cleavage site of *Eco*R I. Green indicates enzyme cleavage site of *Hin*d III.
